# *Salix purpurea* and *Eleocharis obtusa* Rhizospheres Harbor a Diverse Rhizospheric Bacterial Community Characterized by Hydrocarbons Degradation Potentials and Plant Growth-Promoting Properties

**DOI:** 10.3390/plants10101987

**Published:** 2021-09-23

**Authors:** Fahad Alotaibi, Soon-Jae Lee, Marc St-Arnaud, Mohamed Hijri

**Affiliations:** 1 Institut de Recherche en Biologie Végétale, Université de Montréal and Jardin Botanique de Montréal, Montréal, QC H1X 2B2, Canada; fanalotaibi@ksu.edu.sa (F.A.); soon-jae.lee@unil.ch (S.-J.L.); marc.st-arnaud@umontreal.ca (M.S.-A.); 2 Department of Soil Science, King Saud University, Riyadh 11564, Saudi Arabia; 3 African Genome Center, Mohammed VI Polytechnic University (UM6P), Ben Guerir 43150, Morocco

**Keywords:** phytoremediation, petroleum hydrocarbon-degrading bacteria, *Salix*, plant growth promoting rhizobacteria, *Eleocharis*, alkanes, polycyclic aromatic hydrocarbons

## Abstract

Phytoremediation, a method of phytomanagement using the plant holobiont to clean up polluted soils, is particularly effective for degrading organic pollutants. However, the respective contributions of host plants and their associated microbiota within the holobiont to the efficiency of phytoremediation is poorly understood. The identification of plant-associated bacteria capable of efficiently utilizing these compounds as a carbon source while stimulating plant-growth is a keystone for phytomanagement engineering. In this study, we sampled the rhizosphere and the surrounding bulk soil of *Salix* *purpurea* and *Eleocharis obusta* from the site of a former petrochemical plant in Varennes, QC, Canada. Our objectives were to: (i) isolate and identify indigenous bacteria inhabiting these biotopes; (ii) assess the ability of isolated bacteria to utilize alkanes and polycyclic aromatic hydrocarbons (PAHS) as the sole carbon source, and (iii) determine the plant growth-promoting (PGP) potential of the isolates using five key traits. A total of 438 morphologically different bacterial isolates were obtained, purified, preserved and identified through PCR and 16S rRNA gene sequencing. Identified isolates represent 62 genera. Approximately, 32% of bacterial isolates were able to utilize all five different hydrocarbons compounds. Additionally, 5% of tested isolates belonging to genera *Pseudomonas*, *Acinetobacter*, *Serratia*, *Klebsiella*, *Microbacterium, Bacillus* and *Stenotrophomonas* possessed all five of the tested PGP functional traits. This culture collection of diverse, petroleum-hydrocarbon degrading bacteria, with multiple PGP traits, represents a valuable resource for future use in environmental bio- and phyto-technology applications.

## 1. Introduction

Industrial activities such as mining for minerals, oil and gas extraction, inorganic fertilizer-based agriculture, and industrial waste disposal, are all associated with environmental contamination risks which represent a global challenge [1]. Among pollutants, petroleum hydrocarbons (PHCs) are of great concern and can pose a high risk in oil spills, and environmental contamination of aquatic and terrestrial ecosystems. PHCs, like crude oil, are heterogeneous organic mixtures composed of carbon and hydrogen atoms and are broadly classified into two major fractions: (1) aliphatic hydrocarbons, like alkenes, alkynes, or alkanes, and (2) aromatic hydrocarbons, including mono-aromatic (i.e., benzene, toluene, phenol, etc.), and polycyclic aromatic hydrocarbons (PAHs) [2,3]. The main sources of PHCs contamination in the environment are mostly anthropogenic, and include accidental release (i.e., diesel, solvent), and industrial activities (i.e., production of electricity, petrochemical activities) [4]. Environmental contamination with PHCs products has adversely affected various ecosystems, including soils, causing damage to natural habitats with serious economic consequences [5].

Concerns regarding soil pollution with PHCs have initiated the development of several remediation technologies, including biological, chemical and physical methods [2,6]. A promising biological technology for the removal of PHCs from soil is phytoremediation: an eco-friendly, green, solar-driven, and low carbon footprint approach that utilizes plants and their root-associated rhizospheric and endophytic microbiomes to clean-up PHC-contaminated soils [7,8]. Phytoremediation has proven its ability in remediating moderately polluted soils. However, phytoremediation has unreliable effectiveness at high levels of contaminants because of the reduced growth of introduced plants in these conditions. This reduction in plant growth may be partially due to variation in the association between plants and their resident microbiomes [9,10,11,12,13]. 

Over the last decade, most of the research efforts aimed at enhancing the efficiency of phytoremediation of PHCs focused on using plant species that can tolerate high levels of PHCs, such as *Salix* spp. [2,8,10,11,14]. *Salix* spp. (willows), which have been shown to be effective in decontaminating soils polluted with organic compounds, such as PHCs, and trace metals. Willows have several characteristics that may facilitate phytoremediation, including their ease of propagation, fast and perennial growth patterns, high-biomass production, high-contaminants tolerance, and massive deep-root systems [2,15,16,17]. Additionally, several recent studies have shown that *Salix* spp. can recruit certain microbial taxa that could help the plant to cope with PHCs contamination stress and accelerate the biodegradation process [10,18,19,20].

More recently, a promising strategy that includes the screening and identification of native plants growing spontaneously on PHCs-contaminated soils has been adopted [21,22,23,24]. This is the reason we chose *Eleocharis obtusa* (Willd), which dominated the vegetation at the site of study [21]. *Eleocharis* spp. are ubiquitous plants distributed across Canada and United States, where they grow in wetlands. These plants are not used in phytoremediation. Pérez-Jaramillo et al. [25] proposed a “back to the roots” frame that involves the survey of native plants, and their associated microbiomes, in their native habitats, with the goal of restoring plant-microbial associations that may have been diluted during plants domestication [25]. Native plants are more genetically diverse and more adapted to wide-ranging climatic conditions compared to other plant species currently chosen for the phytoremediation of PHCs [26]. Additionally, native plants have been shown to develop more close relationships with local rhizosphere microbiota than introduced plants [11,27], thus making native plants ideal models to study how microbiomes respond to environmental pollutions and explore their future use in the phytoremediation of PHCs. 

The rhizomicrobiome, a subset of the plant holobiont, refers to the soil microbiomes associated with a plant’s roots. The rhizomicrobiome contributes to the functioning of plants including through the removal and degradation of PHCs compounds in contaminated soils [28,29]. Plants growth under stress such as PHCs contamination is expected to be lower than it would be under optimal conditions [30]. However, exploiting the potential of plant growth-promoting rhizobacteria (PGPR) in phytoremediation of PHC-contaminated soils holds great promise as it has recently been demonstrated [31,32,33]. PGPR are soil microbes within the rhizomicrobiome with phenotypes that benefit plant growth [28]. Therefore, plant growth may be positively stimulated by the presence of rhizobacteria with plant growth-promoting (PGP) traits, which alleviate stresses in plants via several mechanisms including: reducing soil nutrient deficiencies (fixing nitrogen, solubilizing phosphorus and enhancing iron uptake), synthesizing plant growth hormones, reduction in ethylene production via 1-aminocyclopropane-1-carboxylate (ACC) deaminase activity, as well as [30,34] degrading a broad range of PHCs compounds [2].

It is well documented that some rhizospheric bacteria have beneficial effects on their host in natural and anthropized terrestrial ecosystems. However, the role of rhizobacteria in association with plants that spontaneously grow in heavily PHC-polluted areas is not widely explored. However, some reports documented the influence of pollutants on microbial community structures [35,36]. The aim of this study was to isolate and characterize the PGPR and hydrocarbon-degraders associated with *Salix purpurea* and *Eleocharis obtusa* plants growing in a long-term petroleum hydrocarbon-polluted petrochemical site. We hypothesized that the rhizosphere of *S. purpurea* and *E. obtusa* plants growing in soils chronically contaminated with PHCs would harbor diverse bacterial communities with multiple key species having hydrocarbon degrading potential and PGP traits.

To address our hypothesis, a structurally and functionally diverse collection of PGPR and degradative bacteria were isolated from the rhizosphere of *Salix* and *Eleocharis* plants collected in the contaminated site. The cultured bacteria were all assessed for their abilities to grow in the presence of alkanes and polycyclic aromatic hydrocarbons as the sole carbon source, as well as for their PGP traits. 

## 2. Results

### 2.1. Isolation and Characterization of Bacteria 

Four hundred and thirty-eight morphologically distinct bacterial isolates were initially selected, purified and preserved in −80 °C. Bacterial isolates were identified based on the Sanger sequencing of their 16S rRNA gene. Among the 438 isolates identified, 146 bacterial isolates were recovered from *S. purpurea* rhizosphere, 146 isolates from *E. obtusa* rhizosphere and 146 isolates from bulk soil, as are shown in Supplementary Tables S1–S3, respectively. 

Bacterial isolates were classified into 62 genera, belonging to the phyla Actinobacteria, Bacteroidetes, Firmicutes and the Alpha, Beta and Gamma-subgroups of Proteobacteria. Interestingly, approximately 5% of the total sequences were not assigned to any known bacteria (Table 1). 

Bacteria from the subphylum Gammaproteobacteria dominated most of the isolates selected, which included 12 genera representing 37.5% of the total sequences. Within this subphylum, the most abundant genera were *Pseudomonas* (14.3%), *Klebsiella* (5%), *Acinetobacter* (4%), *Pseudoxanthomonas* (3.8%), *Enterobacter* (3%), *Stenotrophomonas* (2.7%), *Rheinheimera* (1.3%) and *Serratia* (1.1%) (Table 1).

The phylum Actinobacteria corresponded to 29% of the bacterial isolates, and was represented by 18 genera. The most abundant genera were *Streptomyces* (7.3%), *Microbacterium* (5.2%), *Arthrobacter* (4.5%), *Rhodococcus* (3%), *Nocardioides* (1.8%), *Mycobacterium* (1.3%) and *Gordonia* (1%) (Table 1). The third most predominant phylum was Firmicutes representing 11% of the total bacterial collection, with *Bacillus* (9%) and *Exiguobacterium* (1%) as the dominant genera (Table 1). 

Bacteria from the subphylum Betaproteobacteria correspond to 5% of the total isolates, including seven genera. The two predominant genera were *Variovorax* (3%) and *Massilia* (1%) (Table 1). The sub phylum Alphaproteobacteria correspond to 5% of the total isolates, including 11 genera, with *Rhizobium* (1.1%) as the dominant genus (Table 1). The phylum Bacteroidetes represents 3.5% of the entire bacterial collection (Table 1).

**Figure 1:**
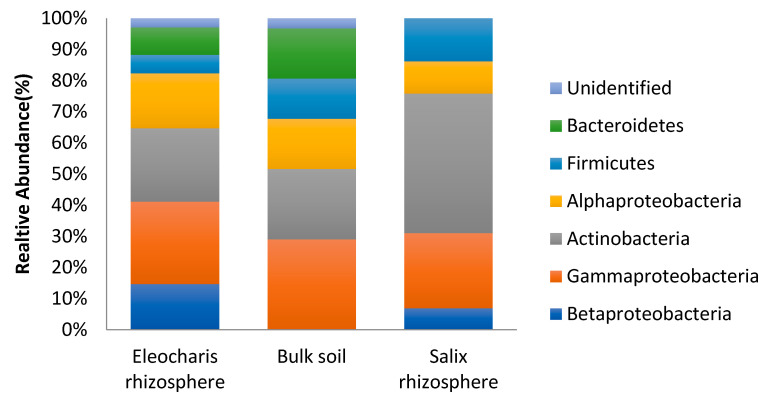
Taxonomic breakdown and relative abundance of bacterial isolates at the phylum level.

The *E. obtusa* rhizosphere was dominated by Gammaproteobacteria, followed by Actinobacteria and Alpha- and Betaproteobacteria, Bacteroidetes, Firmicutes and unidentified bacteria (Figure 1). The *S. purpurea* rhizosphere was dominated by Actinobacteria, followed by Gammaproteobacteria, Firmicutes, Alpha- and Betaproteobacteria (Figure 1). In the polluted bulk soil, Gammaproteobacteria was the predominant phyla, followed by Actinobacteria, Alphaproteobacteria, Bacteroidetes, Firmicutes and unidentified bacteria (Figure 1). 

**Figure 2:**
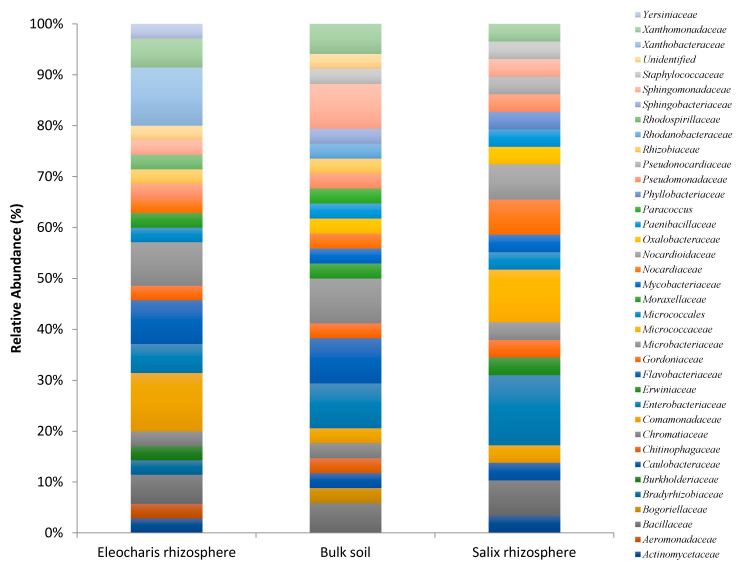
Taxonomic breakdown and relative abundance of bacterial isolates at the family level.

**Figure 3:**
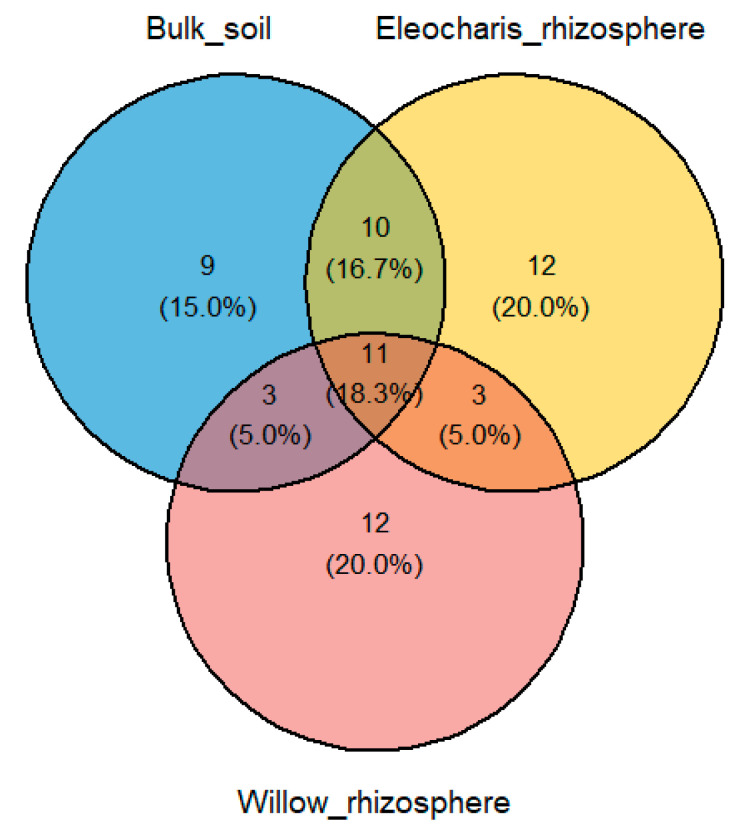
Identity of bacterial isolates at the genus level shared among the three environmental niches.

Dominant families within the E. obtusa rhizosphere were Xanthobacteraceae, Comamonadaceae, Microbacteriaceae, Flavobacteriaceae, Bacillaceae, Xanthomonadaceae and Enterobacteriaceae (Figure 2). S. purpurea rhizosphere was dominat by Enterobacteriaceae, Micrococcaceae, Nocardioidaceae, Nocardiaceae and Bacillaceae (Figure 2). Several families were predominant in the bulk soil including Sphingomonadaceae, Flavobacteriaceae, Enterobacteriaceae, Microbacteriaceae, Bacillaceae and Xanthomonadaceae (Figure 2). Notably, several families with known petroleum hydrocarbons degradation capabilities were present in all the three environmental habitats, including Comamonadaceae, Enterobacteriaceae, Microbacteriaceae, Sphingomonadaceae, Bacillaceae, Xanthomonadaceae, Gordoniaceae and Nocardiaceae (Figure 2). Notably, 11 bacterial genera were shared between the S. purpurea rhizosphere, E. obtusa rhizosphere and the bulk soil (Figure 3).

### 2.2. Petroleum-Hydrocarbon Degradation Potential

**Figure 4:**
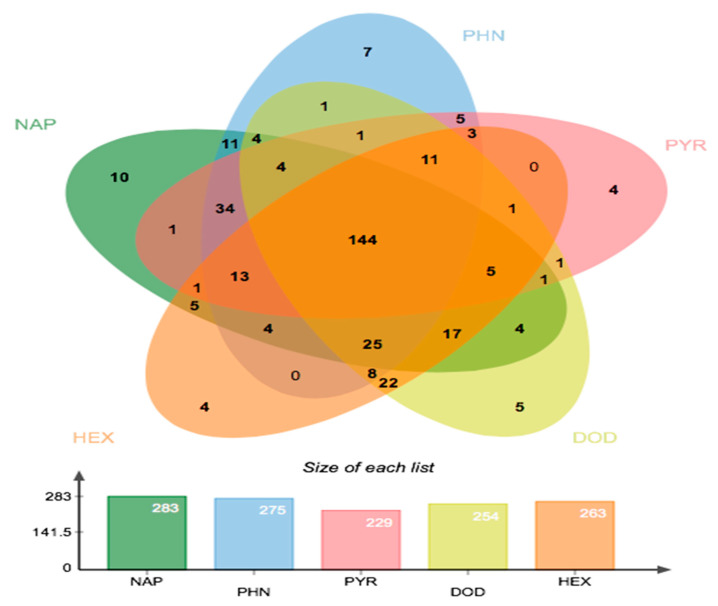
Bacterial isolates that were able to grow with various petroleum hydrocarbons as the only carbon source, showing degradation potential. (Above) Venn-diagram showing the number of isolates that grew on one or many of the five (PHC) compounds. (Below) Bar graph indicating the absolute numbers of bacterial isolates that grew on each of the (PHC) compounds under investigation.

All bacterial isolates were assessed for their ability to degrade various alkanes (*n*-hexadecane and dodecane) and polycyclic aromatic hydrocarbons (PAHs) compounds (naphthalene, phenanthrene and pyrene). Our results indicate that 144 bacterial strains out of the total 438 isolates were able to utilize all 5-hydrocarbon compounds under investigation. Focusing on bacterial isolates able to degrade PAHs, 283 bacterial strains (64%) were able to utilize naphthalene (2-rings PAH-compound) and 275 bacterial strains (62%) were able to utilize phenanthrene (3-rings PAH-compound) (Figure 4). Additionally, 229 bacterial strains (52%) were able to utilize pyrene (4-rings PAH-compound) as a sole carbon source (Figure 4).

Regarding *n*-alkanes degrading bacteria, our results show that 254 bacterial strains (57%) were able to utilize dodecane (12-carbon compound) and 263 bacterial strains (60%) were able to utilize *n*-hexadecane (16-carbon compound) as sole carbon source (Figure 4).

**Figure 5:**
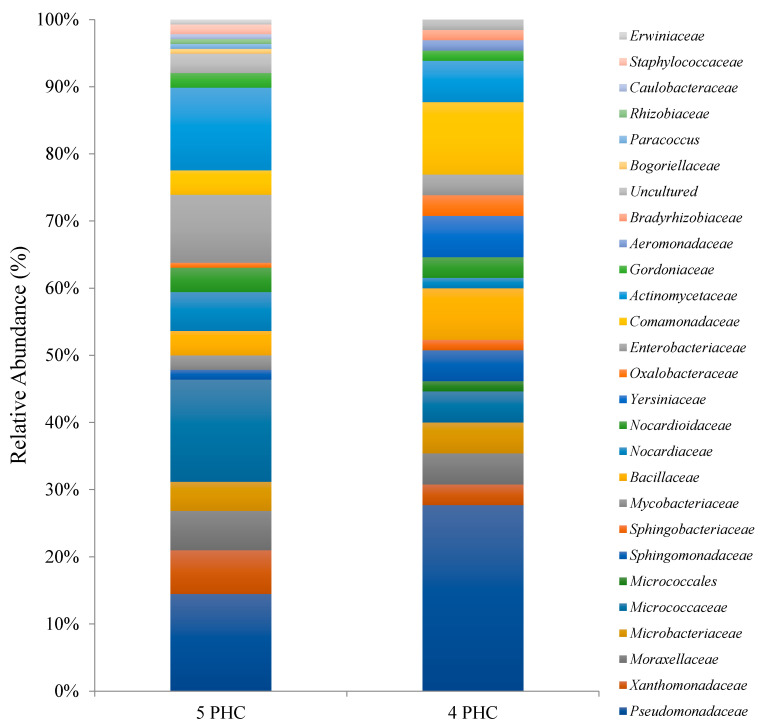
Bars indicate the relative abundance of genera among isolates, which are able to degrade five and four different petroleum hydrocarbons compounds (PHC) in vitro.

About 32% of isolates were able to utilize all 5-hydrocarbon compounds tested in this study (Figure 5). Of those, 16% belonged to the family *Micrococcaceae*, 15% to *Pseudomonadaceae*, 13% to *Actinomycetaceae*, 10% to *Enterobacteriaceae*, 7% to *Xanthomonadaceae*, 5% to *Moraxellaceae*, and 4% to *Microbacteriaceae* (Figure 5).

Isolates that were able to utilize four hydrocarbon compounds represented 41% of total bacterial collection, and 26% of them belonged to the family *Pseudomonadaceae* followed by *Comamonadaceae* (10%), *Bacillaceae* (8%), *Actinomycetaceae* (7%), *Yersiniaceae* (5%), *Microbacteriaceae* (4%), *Micrococcaceae* (4%), *Moraxellaceae* (4%) and *Sphingomonadaceae* (4%) (Figure 5).

### 2.3. Plant Growth-Promoting Traits 

**Figure 6:**
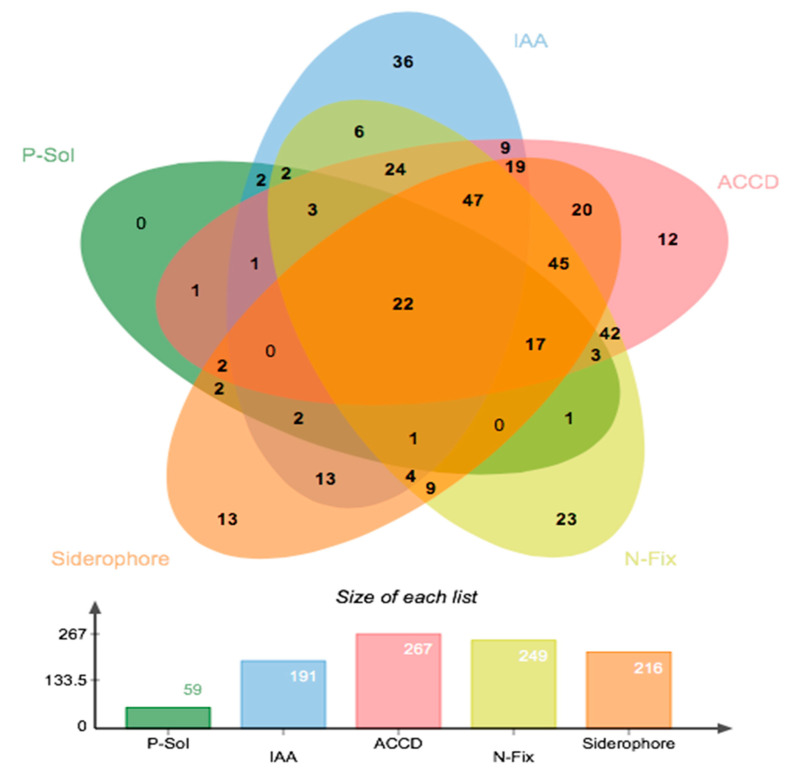
Bacterial isolates with plant growth-promoting (PGP) properties. (Above) Venn diagram showing the number of isolates that possess one or many of the five PGP traits. (Below) Bar graph indicating the absolute numbers of bacterial isolates having each of the PGP traits under investigation (over 438 isolates tested).

Our 438 bacterial isolates were screened for traits that are commonly associated with plant growth-promoting (PGP) abilities. Our results show that only 22 (5%) bacterial isolates were positive for all five PGP traits (Table 2 and Figure 6). Results also show that 267 isolates (60%) were able to grow on a DF-minimal salt medium with ACC as the sole nitrogen source, indicating the presence of ACC deaminase (Figure 6), 249 bacterial isolates (56%) were able to fix nitrogen, 216 bacterial isolates (49%) were able to synthesize siderophores, 191 bacterial isolates (43%) were able to produce IAA and 59 bacterial isolates (13%) could solubilize inorganic phosphorus (Figure 6).

**Figure 7:**
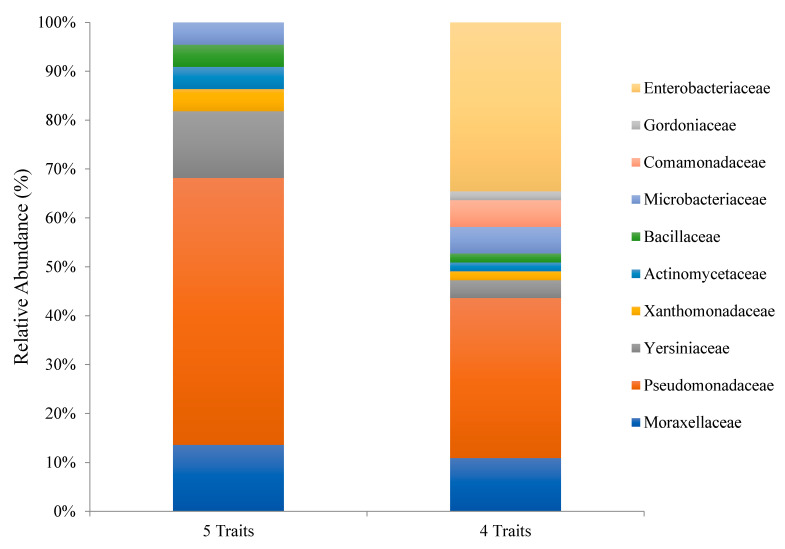
Bars indicate the relative abundance of families among isolates presenting four or five different PGP-associated traits in vitro.

From the 22 isolates (or 5% of all isolates) that were positive for all PGP traits, 45% belonged to the family *Pseudomonadaceae*, 15% to *Moraxellaceae*, 14% to *Yersiniaceae*, 5% to *Microbacteriaceae*, 4% to *Bacillaceae*, 3% to *Actinomycetaceae* and 3% to *Xanthomonadaceae* (Figure 7).

Isolates that were positive for at least four PGP traits represented (12%) of total bacterial collection, with *Pseudomonadaceae* being the predominant family being (33%) followed by *Enterobacteriaceae* (32%), *Moraxellaceae* (12%), *Comamonadaceae* (5%) and *Microbacteriaceae* (4%) (Figure 7). 

## 3. Discussion

The study of rhizosphere microbial communities associated with plants growing in long-term PHC-contaminated soil represents an opportunity for phytoremediation research. Several reports described the microbial community structures, diversities and functions in the rhizosphere of planted *Salix* trees as well as in ruderal plants growing spontaneously in soils highly contaminated with PHCs, using different sequencing techniques including cloning [37,38], next generation-targeted amplicon sequencing [10,11,20] and metatranscriptomics [14,39]. This study used a conventional microbiological approach to isolate, identify and characterize bacteria with multiple petroleum hydrocarbon-degrading capacities and plant growth-promoting capabilities to generate a bacterial culture collection for future use as a source of bacterial inoculants to enhance phytoremediation of PHCs-contaminated soils. 

High concentrations of PHCs cause phytotoxic effects on plants growing in contaminated soils [23,40]. For example, the growth rates of corn and red bean plants were reduced at 10000 mg/kg of crude oil [40]. Similarly, Chaîneau et al. [41] reported a stunted plant growth and inhibitory effects on the seed germination of several plants such as *Helianthus annuus*, *Zea mays, Lactuca sativa, Phaseolus vulgaris, Triticum* sp. and *Trifolium* sp. when exposed to high concentrations of fuel oil ranging from 3000 to 12,000 mg/kg. However, despite the devastating effects of PHCs on plant growth, recent studies have reported that several spontaneously growing herbaceous plants were found to flourish in highly contaminated soils near abandoned oil wells where the concentrations of PHCs could reach up to 45,000 mg/kg [22,42]. Similarly, Desjardins et al. [21] reported three indigenous plant species (*Alisma triviale*, *Eleocharis obtuse* and *Panicum capillare*) that grow spontaneously in highly petroleum-contaminated decantation basins of a former petrochemical plant in Varennes (southern Québec, Canada). These plants were tolerant of high levels of PHCs where the concentrations could reach up to 26300 mg/kg [21]. Moreover, not only spontaneously growing indigenous plants could tolerate high concentrations of PHCs, but also introduced pioneering phytoremediator plants such as *Salix* sp. were able to tolerate such a high level of PHCs [10,14,20].

The results of this study support our hypothesis that plants growing in soil chronically contaminated with PHCs would select for rhizospheric bacteria with multiple petroleum hydrocarbon-degrading potential and plant growth-promoting capabilities. In this study, 438 bacterial strains were isolated from bulk soil, and the rhizosphere soil of *S. purpurea* and *E. obtuse* rhizosphere soil using three different isolation strategies to enhance the diversity of bacterial isolates with multiple petroleum-hydrocarbon degradation potentials and plant growth-promoting traits. Our isolation strategies resulted in a culture collection of bacterial strains belonging to Actinobacteria, Alpha- Beta- and Gammaproteobacteria, Bacteroidetes and Firmicutes (Figure 1), encompassing a fairly diverse collection of bacterial genera (62 genera) (Table 1), including *Acinetobacter, Arthrobacter, Bacillus, Chitinimonas, Enterobacter, Gordonia, Klebsiella, Microbacterium, Mycobacterium, Nocardia, Nocardioides, Pseudomonas, Pseudoxanthomonas, Rhodococcus, Serratia, Sphingomonas, Stenotrophomonas, Streptomyces* and *Variovorax* (Table 1). Several of these genera have previously been shown to hold promising petroleum-hydrocarbons degradation potential and plant growth-promoting activities [30,43,44,45,46]. The selected media used in our study did not result in the cultivation of new phyla; however, expandable bacterial culture collections could be established using additional novel cultivation strategies, as previously demonstrated for *Arabidopsis thaliana At*-SPHERE culture collection [47].

Our study revealed that culturable rhizospheric bacteria associated with *S. purpurea* rhizosphere mainly belonged to Actinobacteria and Gammaproteobacteria (Figure 1). In contrast to our results, Bell et al. [10] studied the bacterial community structure and composition in the rhizosphere of several willows cultivar growing in PHCs-contaminated soils using 454-pyrosequencing and found that Betaproteobacteria was the predominant phyla. One possible explanation for this result is that a selective medium was used in this study (Bushnell-Haas medium amended with 1% diesel) to isolate PHC-degrading bacteria, while in Bell et al. [10], all bacteria were potentially amplified and sequenced. In agreement with our explanation, Ferrera-Rodríguez et al. [48] reported that culturable rhizospheric bacteria from five Arctic native plant species growing in PHC-contaminated soils were similarly dominated by Actinobacteria and Gammaproteobacteria when a selective medium was used to isolate PHC-degrading bacteria. The predominant family within the willow rhizosphere was *Enterobacteriaceae* (Figure 2). Recent studies have reported that genera belonging to the family *Enterobacteriaceae* were predominant in the root endosphere of plants growing in Athabasca oil sands reclamation sites [49] and herbaceous plants growing near natural oil seep fields [50]. Endophytic bacteria are thought to be a subset of the larger rhizosphere microbiota [34] and further studies looking at the composition of culturable endophytic bacteria of *Salix* plants growing in PHC-contaminated soils will be required in order to elucidate the role of bacterial endophytes to improve PHC-phytoremediation. Other predominant families included *Micrococcaceae, Nocardioidaceae and Nocardiaceae* (Figure 2), which have been shown to possess strong petroleum hydrocarbon degradation capabilities [45,46].

To our knowledge, there are no other reports concerning the isolation and identification of rhizospheric bacteria from *E. obusta*. Our study revealed that culturable rhizospheric bacteria associated with the *E. obusta* rhizosphere were mainly affiliated to Gammaproteobacteria, Actinobacteria and Betaproteobacteria phyla (Figure 1). The Dominant families were *Comamonadaceae, Xanthomonadaceae* and *Microbacteriaceae* (Figure 2). Comamonad bacteria (phylum Betaproteobacteria), for instance, are known to contain genera such as *Comamonas*, *Delftia* and *Variovorax*, which exhibit an extraordinary capability of degrading wide spectra of PHCs [45,46]. Genera belonging to the phyla Gammaproteobacteria, Actinobacteria are also known to contain bacterial species with efficient petroleum hydrocarbon degradation potentials [45,46] such as *Pseudomonas*, *Streptomyces* and *Rhodococcus* [51,52].

Soil and rhizospheric bacteria can increase the phytoremediation of PHCs by decreasing the level of PHCs in the contaminated soils via their enzymatic machinery mostly under aerobic conditions [53]. The results obtained in our study indicate that many bacterial isolates originating from the contaminated soil and rhizosphere samples have the potential to degrade a wide range of PHC compounds. More than 32% of our bacterial isolates were able to degrade all PHC being tested (Figure 4). Petroleum hydrocarbon-degrading bacteria isolated in this study belonged mainly to Actinobacteria (mostly *Streptomyces*, *Arthrobacter*, *Rhodococcus* and *Nocardia*), Proteobacteria (mostly *Pseudomonas*, *Enterobacter*, *Stenotrophomonas*, *Acinetobacter* and *Variovorax*) and Firmicutes (mostly *Bacillus*). Previous reports have shown that many bacterial genera belonging to these phyla were able to degrade a wide range of PHC compounds [36,46,54]. For example, the genus *Rhodococcus* has demonstrated high efficiency in degrading and transforming a wide range of organic substances, including aliphatic and aromatic hydrocarbons, pesticides and petroleum [55,56]. Therefore, there are immense interests in utilizing *Rhodococcus* in bioremediation of polluted soils due to their safe and ease of culturing and maintenance, and high catabolic *versatility* [52,55,56].

Bacterial isolates with PGP traits provide critical functions for their host plants growing in stressful environments, such as soil contaminated with PHCs. Isolating bacteria from PHC-contaminated environments that have both PGP traits and PHC-degrading activities has been of great interest in a new paradigm of environmental cleanup biotechnology which exploits PGPR. Selecting plants suitable for phytoremediation depends on many criteria, the most important of which is root morphology [15]. PGPR with the capacity to produce the phytohormones IAA, which plays a role in inducing the formation of lateral roots [43], would further stimulate plant growth in PHC-contaminated soils. In this study, 43% of bacterial isolates synthesized IAA (Figure S1), which were mostly affiliated to the genera *Pseudomonas, Streptomyces, Enterobacter, Arthrobacter and Microbacterium* (Figure S2). Previous studies confirmed that IAA-producing genera reported in this work were also found to produce IAA by endophytic and rhizospheric bacteria isolated from various plants [24,57,58]. 

Another mechanism by which PGPR have the potential to improve plant growth under adverse environmental conditions, including PHC contamination, is by producing the enzyme ACC deaminase [30,43]. Stressed plants induce the production of the phytohormone ethylene to bolster their defense. However, ethylene also inhibits plant growth [59]. Certain PGPR can inhibit ethylene biosynthesis via the production of ACC deaminase which cleaves the ethylene precursor ACC into alpha-ketobutyrate and ammonia [30,59]. In this study, 60% of bacterial isolates were found to produce ACC deaminase (Figure S1). Most isolates that could catabolize ACC reported in this work belonged to genera such as *Pseudomonas*, *Klebsiella, Enterobacter, Stenotrophomonas and Microbacterium* (Figure S2). The high percentage of ACC deaminase-producing bacteria among our isolates corroborate previous studies reporting the widespread nature of this trait in various soil bacteria [60,61,62]. 

N fixation, phosphate solubilization, and siderophore production are some of the direct PGP mechanisms making nutrients available to plants. These traits were found among the bacteria isolated of this study (Figure S1). Nitrogen fixation by diazotrophic bacteria is an important trait of PGPR that benefits the plant, especially when growing in nutrients-deficient soils [63]. Diazotrophic bacteria isolated in this study belonged mainly to genera such as *Pseudomonas*, *Klebsiella, Bacillus*, *Enterobacter*, *Acinetobacter* and *Variovorax* (Figure S2). Low levels of soluble P in soils can restrict the growth and development of plants [43]. Some PGPR solubilize inorganic forms of P and convert it to plant-available forms, thereby facilitating plant growth [64,65]. Our study found that the majority of isolates are able to solubilize inorganic P belonged to the genera *Pseudomonas*, *Acinetobacter*, *Bacillus* and *Serratia* (Figure S2). Another essential nutrient for plant growth is iron, even if it is present in soils in the highly insoluble form Fe^3+^ [66]. Some PGPR produce low molecular-weight organic compounds, siderophores, that chelate Fe^3+^ ions and render them available for reduction to the soluble Fe^2+^ form preferred by plants [66]. The majority of isolates reported in this study that were able to produce siderophores belonged to the genera *Pseudomonas*, *Acinetobacter, Microbacterium, Rhodococcus* and *Stenotrophomonas* (Figure S2). The widespread ability of our isolates to hold PGP traits related to increasing the concentration and availability of nutrients to plants is of great importance to the plant nutrition balance. 

This study highlights the functional potential of this culture collection in which many bacterial isolates, from the genera *Acinetobacter, Arthrobacter, Nocardia, Rhodococcus, Streptomyces* and *Variovorax*, possessed petroleum hydrocarbon degradation capabilities. However, only a small proportion of bacterial isolates (5%) had multiple PGP traits. These strains were isolated from the genera *Acinetobacter*, *Enterobacter*, *Klebsiella*, *Pseudomonas* and *Serratia*. Interestingly, in our study, only three bacterial isolates were capable of degrading all five PHCs, and had all five PGP traits (Figures S3–S5): *Pseudomonas putida* strain EB3, *Streptomyces* sp. strain WT8 and *Bacillus* sp. strain WT32. These findings corroborate earlier studies which reported that many isolates from these genera can degrade PHCs and promote plant growth [51,67,68]. These bacterial taxa are candidates to look for in follow-up experiments. 

## 4. Materials and Methods

### 4.1. Site Description, Experimental Design and Sample Collection

Soil samples were collected from *Salix purpurea* L. cv “Fish Creek” and *Eleocharis obtusa* (Willd.) Schult. plants growing on a former petrochemical plant located on the south shore of the St-Lawrence River in Varennes, Québec, Canada (45°43′ N, 73°22′ W) (for details on the site, see Bell et al. [10] and Desjardins et al. [21]). The petrochemical plant was fully operated from 1953 until it was closed in 2008 [69]. The soil was contaminated with a mixture of alkanes and PAHs. Previous studies have analyzed contaminated soil samples from the site for F1-F4 hydrocarbons fractions (the sum of aliphatic and aromatic compounds with chain lengths of C6–C50). Analysis showed that the soil contamination was variable but reached concentrations averaging 3590 mg kg−1 [10], which exceeds by far the limit for land reuse defined by the government of Québec for industrial areas.

About 10,000 trees of eleven different *Salix* cultivars were planted in the contaminated soil in a split-plot design in this site in 2011, as part of a large phytoremediation pilot project (see Bell et al. [10] for details), while *E. obtusa* plants began spontaneously growing in the polluted soil across the site. We took advantage of this larger design to sample five four-year-old *S. purpurea* trees and five fully-grown *E. obtusa* plants, which were randomly selected from the site on 13 August 2015. *S.* *purpurea* *and E. obtusa* plants were dug out and shaken vigorously to dislodge the bulk soil attached to the roots; only the soil that remained strongly adhered to the roots (i.e., rhizosphere soil) was collected, from a zone of the root system growing approximately 0 to 15 cm in depth from the surface. Approximately 50 g of rhizospheric soil was collected from each plant, while five bulk soil samples free of any plant materials were randomly collected from the site as bulk samples. Bulk soil samples were taken from the top 15 cm of soil at least 50 cm from the nearest plants. Rhizosphere and bulk soil samples were placed in sterile Whirl-Pack^®^ bags (Sigma-Aldrich, Oakville, ON, Canada) and put on ice until transportation to the laboratory. 

A composite sample for chemical analysis was formed from each of the five *S. purpurea* rhizosphere soil samples, *E. obtusa* rhizosphere soil samples, and bulk soil samples. Chemical analysis showed that the soil samples had high Total Petroleum Hydrocarbons (TPH) concentrations. The mean TPH concentrations were 10000 mg/kg for the bulk soil samples, 4800 mg/kg for the *E. obtusa* rhizosphere samples and 1400 mg/kg for the *S. purpurea* rhizosphere samples. Other soil chemical and physical parameters are listed in Table 3.

### 4.2. Bacterial Isolation 

Three different growing media were used to isolate the bacteria from the soil samples: (1) Bushnell-Haas medium amended with 1% diesel, as the sole carbon source; (2) one-tenth-strength Trypticase Soy Agar (TSA) medium; and (3) Dworkin & Foster (DF) minimal salts medium containing ACC. These media were used for the isolation and cultivation of petroleum hydrocarbon-degrading bacteria, total heterotrophic bacteria and ACC deaminase-producing PGPR, respectively. 

Bushnell-Haas agarose plates amended with 1% diesel were prepared as follows (per liter): 0.2 g MgSO_4_, 0.020 g CaCl_2_, 1 g KH_2_PO_4_, 1 g K_2_HPO_4_, 1 g NH_4_NO_3_, 0.050 g FeCl_3_, 17 g agarose; the final pH was adjusted to 7 and the medium was sterilized by autoclaving at 121 °C for 25 min [70]. One percent filter-sterilized (0.2 µm pore size membrane) diesel was added to the Bushnell-Hass medium before pouring the plates. One-tenth-strength TSA plates were prepared by suspending 3 g trypticase soy broth (Difco Laboratories, Detroit, MI, USA) and 15 agar (Difco Laboratories, Detroit, MI, USA) per L of distilled water before sterilizing the medium by autoclaving it at 121 °C for 25 min. The DF minimal salts agar plates were prepared as follows (Dworkin and Foster 1958) (per liter): 4 g KH_2_PO_4_, 6 g Na_2_HPO_4_, 0.2 g MgSO_4_·7H2O, 2.0 g glucose, 2 g gluconic acid, 2 g citric acid, 0.1 mL of trace elements solution (10 mg H_3_BO_3_, 11.19 mg MnSO_4_·H_2_O, 124.6 mg ZnSO_4_·7H_2_O, 78.22 mg CuSO_4_·5H_2_O, and 10 mg MoO_3_), 0.1 mL of FeSO_4_·7H_2_O solution and 1.8% Bacto-Agar (Difco Laboratories, Detroit, MI, USA). The pH was adjusted to 7.2 and the medium was sterilized by autoclaving at 121 °C for 25 min. To suppress fungal growth, filter-sterilized cycloheximide (100 mg L^−1^) was added to all three media after autoclaving and just before pouring plates.

For the isolation of petroleum hydrocarbon-degrading bacteria and total heterotrophic bacteria, 5 g of rhizosphere or bulk soil samples were suspended in 95 mL of sterile phosphate buffered saline (PBS; Difco Laboratories, Detroit, MI, USA) and shaken on a rotary shaker (150 rpm) for 30 min. Suspensions were serially diluted in 10-fold series in PBS and 100 μL of the appropriate dilutions (10^−4^ for B-H plates and 10^−5^ for 1/10 TSA plates) were spread in triplicate onto both the Bushnell-Haas agarose plates amended with 1% diesel and on the 1/10 strength TSA plates. The Bushnell-Hass plates and 1/10 TSA plates were incubated at 28 °C for 14 days or 3 days, respectively [71,72]. 

For the isolation of ACC deaminase-producing PGPR, an ACC deaminase enrichment culture method was used as described by Penrose and Glick [73]. Briefly, 1 g of rhizosphere or bulk soil samples were added to 50 mL of sterile Pseudomonas Agar F (PAF) medium containing the following (per liter): 10 g proteose peptone, 10 g casein hydrolysate, 1.5 g anhydrous MgSO_4_, 1.5 g K_2_HPO_4_ and 10 mL glycerol. The culture was incubated in a rotary shaker (200 rpm) at 28 °C for 24 h and a 1-mL aliquot was transferred into a fresh 50-mL sterile PAF medium and incubated under the same environmental conditions. After 24 h, a 1-mL aliquot was transferred into 50-mL sterile DF salts minimal broth medium as described above, except that agar was omitted and 2 g of (NH_4_)_2_SO_4_ was added as a nitrogen source. The culture was incubated in a rotary shaker (200 rpm) at 28 °C for 24 h and a 1-mL aliquot was transferred into a fresh 50-mL sterile DF salts minimal broth medium containing 3 mM filter-sterilized ACC (instead of (NH_4_)_2_SO_4_) as a nitrogen source, and the culture was incubated under the same environmental conditions. After 24 h, 10-fold serial dilutions in PBS were made and 100 μL of the 10^−4^ dilution spread in triplicate onto solid DF salts minimal agar plates amended with ACC (30 μmol plate ^−1^). The solid DF salts minimal agar plates were incubated for 72 h at 28 °C. Colonies showing growth on the plates indicate ACC deaminase production.

Discrete colonies with a distinctive morphology (shapes, size, colors, etc.) were further sub-cultured in order to obtain pure cultures. Isolates were streaked twice on the original medium and checked for purity. Purified isolates were stored in a 1:1 mixture of half-strength Trypticase Soy Broth (TSB) (Difco Laboratories, Detroit, MI, USA) and 20% glycerol (*v*/*v*) and frozen at −80 °C. A total of 438 isolates were collected and further characterized in this study.

Isolated bacterial strains were named based on the medium used for isolation and the rhizosphere zone of origin, and the arbitrary serial number of the strain (i.e., WT15 for the 15th isolate from willow rhizosphere using TSA plates.

### 4.3. DNA Extraction, PCR Amplification, and Sequencing of Bacterial Isolates

A single colony from each purified isolate was inoculated into 5 mL of 1/10 TSB culture media and grown at 28 °C on a gyratory shaker (150 rpm) for 1–3 days until became turbid. Once the liquid culture was ready, an aliquot of 1.8 mL was used to extract genomic DNA using the DNeasy UltraClean Microbial Kit (Qiagen, Toronto, ON, Canada) following the manufacturer’s protocols. 

Isolated genomic DNA was used as a template for the amplification of bacterial 16S rRNA gene by PCR using the primer pair 27F (′5-AGAGTTTGATCMTGGCTCAG-3′) [74] and 1492R (5′-GGTTACCTTGTTACGACTT-3′) [75]. PCRs reactions were performed in 50 μL consisting of 1X PCR Buffer (Qiagen, Toronto, ON, Canada), 0.2 μM each primer, 0.5 mM of MgCl2, 0.2 mM of dNTP mix, 0.2 mg mL_−1_ of BSA (Amersham Biosciences, Mississauga, ON, Canada), 1.25 U of *Taq* DNA polymerase (Qiagen, Toronto, ON, Canada) and 50 ng of gDNA. Thermal cycling conditions were as follows: initial denaturation at 94 °C for 5 min; 30 cycles at 94 °C for 1 min, 55 °C for 1 min, 72 °C for 1 min, and a final elongation at 72 °C for 7 min. PCR products were visualized on GelRed-stained 1.5% agarose gels using the Gel-Doc system (Bio-Rad Laboratories, Mississauga, ON, Canada). DNA sequencing was performed on an Applied Biosystems 3730xl DNA analyzer (Applied Biosystems, Carlsbad, CA, USA) at Génome Québec (Montréal, Canada) [76]. 

### 4.4. Nucleotide Sequence Analyses and Accession Numbers

Sequences obtained from Sanger sequencing were trimmed by removing ambiguous nucleotide sequences, and a pair of forward and reverse reads of the 16S rRNA target was assembled by Geneious Pro v.6.1.5 (Biomatters Inc., San Diego, CA, USA). Bacteria isolates were identified by comparison with reference 16S rRNA genes from GenBank database using the BLAST algorithm [77].

The partial 16S rRNA gene sequences obtained from the bacterial isolates have been deposited in GenBank under the accession numbers (MZ430069-MZ430506). 

### 4.5. Assessment of Hydrocarbon Degradation Potential of the Bacterial Isolates 

Bacterial isolates were assessed for their ability to grow on a Bushnell Haas (BH) mineral salts medium containing various alkanes (*n*-hexadecane and dodecane) and (PAH) compounds (naphthalene, phenanthrene and pyrene) as the sole carbon source as described by Phillips et al. [78]. 

For the alkanes screening, the following ingredients were added into separate wells of a 48-well microtitre plate: 720 μL sterile BH medium and 20 μL of each filter-sterilized hydrocarbon (*n*-hexadecane or dodecane), as the sole carbon source, followed by an addition of 20 μL bacterial suspension of each isolate. Two negative controls were included in the experimental setup: (1) wells containing alkanes and BH without bacterial inoculum, and (2) wells containing alkanes and BH with an autoclaved bacterial culture. After two weeks, 200 μL of filter-sterilized p-iodonitrotetrazolium violet (INT) (3 g L^−1^) (Sigma–Aldrich, Oakville, ON, Canada) was added to each well of the plates, which were then incubated overnight. Wells that were positive for alkane degradation were identified due to the appearance of a red precipitate as a result of the INT reduction to an insoluble formazan that deposits intracellularly [79]. The experiments were repeated twice, each with three replicates for each bacterial isolate.

To screen for PAHs (naphthalene, phenanthrene and pyrene) degradation potential, each PAH compound, of at least 98% purity (Sigma–Aldrich, Oakville, ON, Canada), was first diluted in pentane (5 g L^−1^) (Sigma–Aldrich, Oakville, ON, Canada) and 80 μL of naphthalene, phenanthrene, or pyrene, was added to each well of a 48-well plate, as the sole carbon source, and the pentane was allowed to evaporate. Then, 720 μL sterile BH medium was added to each well, followed by the addition of 20 μL suspension of each bacterial isolate. Two negative controls were included in the experimental setup: (1) wells containing PAHS and BH but no bacterial inoculum, and (2) wells containing PAHS and BH with an autoclaved bacterial culture. PAH plates were incubated for two weeks before 200 μL of filter-sterilized INT (3 g L^−1^) (Sigma–Aldrich, Oakville, ON, Canada) was added to each well. The plates were incubated for an additional week before wells were scored positive for PAH degradation by the presence of a yellow-brown color due to the partial oxidation of aromatic compounds [79]. The experiments were repeated twice, each with three replicates for each bacterial isolate.

### 4.6. Assessment of Plant Growth-Promoting (PGP) Traits of the Bacterial Isolates

#### 4.6.1. Phosphate Solubilization

The ability of bacterial isolates to solubilize inorganic phosphate was assessed using a potato-dextrose yeast agar (PDYA, pH 7.0) medium containing freshly precipitated calcium phosphate [80]. The PDYA medium was prepared in three separate solutions including PDYA-calcium phosphate (CaP) as described by de Freitas et al. [80]. Bacterial cultures were grown in half-strength TSB medium at 28 °C for 48 h with continuous agitation at 150 rpm in a rotary shaker. A loopful of each bacterial isolate growing in liquid culture was streaked in the center of PDYA-calcium phosphate (CaP) plates, and incubated at 28 °C. The appearances of clear zones around colonies were considered as positive phosphate solublizers and measured after 14 days of incubation. An autoclaved bacterial culture was used as a negative control. The experiment was repeated twice, each with three replicates for each isolate.

#### 4.6.2. Screening for Nitrogen Fixation

The bacterial isolates were evaluated for their ability to grow on an N-deficient combined carbon medium which was prepared in two solutions as described by Rennie [81]. Bacterial cultures were grown in half-strength TSB at 28 °C for 48 h with continuous agitation at 150 rpm in a rotary shaker, and a loopful of each bacterial isolate growing in liquid culture was streaked into the N-deficient combined carbon medium agar plate and incubated at 28 °C for up to one week. The formation of colonies on agar plates was considered as positive N-fixers. An autoclaved bacterial culture was used as a negative control. The experiments were repeated twice, each with three replicates for each isolate.

#### 4.6.3. ACC Deaminase Activity

1-Aminocyclopropane-1-carboxylate (ACC)-deaminase activity was assessed by mentoring bacterial isolates’ ability to grow on DF minimal salts medium containing ACC as a sole nitrogen source. The DF minimal salts agar plates were prepared as described above and were spread with filter-sterilized ACC (30 μmol plate^−1^) [73]. Bacterial cultures were grown in half-strength TSB at 28 °C for 48 h with continuous agitation at 150 rpm in a rotary shaker. A loopful of each bacterial isolate growing in liquid culture was streaked into DF minimal salts agar plates containing fresh ACC, which was just spread into the agar plate prior to use. The solid DF salts minimal agar plates were incubated for 72 h at 28 °C. Colonies showing growth on the plates indicate ACC deaminase production. An autoclaved bacterial culture was used as a negative control. The experiments were repeated twice, each with three replicates for each isolate.

#### 4.6.4. Indole-3- Acetic Acid (IAA) Production

The bacterial isolates were screened for the production of the auxin IAA by using the Salkowski colorimetric assay as originally described by Bric et al. [82] and modified by Ribeiro et al. [83]. Isolates were cultured in 15-mL Falcon tubes containing 3 mL of Luria Bertani (LB) medium supplemented with tryptophan (1 mg mL_−1_) as an auxin precursor. Bacterial isolates were grown in a shaker (120 rpm) for 1 day at 28 °C. A 1-mL aliquot of bacterial cultures was then centrifuged at 9500× g for 2 min and 100 μL of supernatant were added to 96 micro-titre plate wells followed by the addition of 100 μL of Salkowski’s reagent (150 mL of 98% H_2_SO_4_, 7.5 mL of 0.5 M FeCl_3_·6H_2_O, and 250 mL distilled water) and the 96 micro-plate was incubated in the dark for 30 min at room temperature. Bacterial isolates producing IAA were characterized by the formation of a distinct red color [82]. An autoclaved bacterial culture was used as a negative control. The experiments were repeated twice, each with three replicates for each isolate.

#### 4.6.5. Siderophore Production

The complex Chrome Azurol S (CAS) solid medium was used to detect siderophore synthesis by the bacterial isolates, as described by Alexander and Zuberer [84]. The assay was performed in 12-well microtitre plates and utilized the ternary complex CAS as an indicator. A change of the color of the indicator from blue to orange designates siderophore production. The CAS-agar medium consists of four solutions as described by Alexander and Zuberer [84]. The CAS-agar medium is poured into 12-microtitre plates by dispensing 5 mL of medium into each well of the plate aseptically. Plates were allowed to solidify before inoculation. Bacterial cultures were grown in half-strength TSB at 28 °C for 48 h with continuous agitation at 150 rpm in a rotary shaker, and 10 μL of liquid bacterial culture was spotted into each well of the micro-titer plate containing the solidified CAS-agar medium. The well plates were incubated at room temperature for 72 h, and the development of an orange-yellow color in the wells indicated siderophore production. An autoclaved bacterial culture was used as a negative control. The experiments were repeated twice, each with three replicates for each isolate.

## 5. Conclusions

*S. purpurea* and *E. obusta* are widespread, native plants in North America, distributed in various habitats and ecosystems, and are able to tolerate chronic levels of PHC pollution. Thus, they are ideal candidates for phytoremediation of PHC-contaminated soils. This culture collection holds 438 bacterial isolates with multiple degradative and PGP features, originating from unique soil environments characterized by high levels of PHC contamination. The functional potential of bacterial isolates reported here represents a rich reservoir of metabolically versatile PGPR-PHC degraders that could be used in holistic, bacterial-aided phytomanagement and remediation of PHC contamination in future research.

## Figures and Tables

**Table 1:** Number of isolates belonging to each bacterial genus associated with *S. purpurea* rhizosphere, *E. obtusa* rhizosphere and bulk soil samples.

Serial #	*Genus* ^1^	*Eleocharis* Rhizosphere ^2^	Bulk Soil ^2^	*Salix* Rhizosphere ^2^
1	*Acidovorax*	1	0	0
2	*Acinetobacter*	3	15	0
3	*Aeromonas*	4	0	0
4	*Agrococcus*	0	1	0
5	*Agromyces*	2	3	0
6	*Amycolatopsis*	0	0	2
7	*Ancylobacter*	1	0	0
8	*Arthrobacter*	0	0	20
9	*Azorhizobium*	2	0	0
10	*Bacillus*	8	16	17
11	*Bosea*	2	0	0
12	*Brevibacillus*	0	1	0
13	*Brevundimonas*	0	2	0
14	*Caulobacter*	0	0	1
15	*Chitinimonas*	2	0	9
16	*Chryseobacterium*	2	2	0
17	*Citrobacter*	0	2	1
18	*Comamonas*	1	0	0
19	*Delftia*	3	0	0
20	*Dyella*	1	3	0
21	*Empedobacter*	1	2	0
22	*Enterobacter*	1	3	3
23	*Exiguobacterium*	2	2	0
24	*Flavihumibacter*	0	1	0
25	*Flavobacteriaceae*	1	0	0
26	*Georgenia*	0	1	0
27	*Gordonia*	1	1	3
28	*Hydrogenophaga*	0	1	0
29	*Klebsiella*	11	7	3
30	*Luteibacter*	0	1	0
31	*Lysinibacillus*	0	0	1
32	*Lysinimonas*	2	0	0
33	*Massilia*	0	4	1
34	*Mesorhizobium*	0	0	1
35	*Microbacterium*	13	9	1
36	*Micromonospora*	0	0	2
37	*Mycobacterium*	1	0	0
38	*Mycolicibacterium*	2	3	2
39	*Myroides*	3	2	0
40	*Nocardia*	0	0	2
41	*Nocardioides*	0	0	9
42	*Paenarthrobacter*	0	0	2
43	*Paenibacillus*	0	0	1
44	*Pantoea*	1	1	1
45	*Phycicoccus*	0	0	2
46	*Pseudarthrobacter*	0	0	2
47	*Pseudomonas*	36	11	15
48	*Pseudoxanthomonas*	5	12	0
49	*Raoultella*	0	0	6
50	*Rheinheimera*	1	2	0
51	*Rhizobium*	4	1	0
52	*Rhodococcus*	2	6	2
53	*Rhodospirillum*	1	0	0
54	*Serratia*	5	0	0
55	*Sphingobacterium*	0	2	0
56	*Sphingobium*	0	1	0
57	*Sphingomonas*	1	1	1
58	*Sphingopyxis*	2	1	0
59	*Staphylococcus*	0	1	1
60	*Stenotrophomonas*	3	8	1
61	*Streptomyces*	1	0	32
62	*Unidentified bacteria*	7	14	0
63	*Variovorax*	1	0	12

^1^ Closest identity at the genus level of our sequences using BLAST in the Genbank database. ^2^ Number of bacterial isolates obtained per genus.

**Table 2:** List of bacterial isolates that possessed all five-plant growth-promoting traits that were tested in this study.

Serial #	Isolate	Closest NCBI Relative	Environmental Niche	Isolation Medium
1	SB41	*Acinetobacter calcoaceticus*	Bulk soil	B-H_amended diesel
2	SB55	*Acinetobacter calcoaceticus*	Bulk soil	B-H_amended diesel
3	SB60	*Acinetobacter* sp.	Bulk soil	B-H_amended diesel
4	ET27	*Pseudomonas plecoglossicida*	*Eleocharis* rhizosphere	TSA
5	ET43	*Serratia* sp.	*Eleocharis* rhizosphere	TSA
6	ET45	*Pseudomonas fluorescens*	*Eleocharis* rhizosphere	TSA
7	ET46	*Serratia* sp.	*Eleocharis* rhizosphere	TSA
8	ET50	*Pseudomonas putida*	*Eleocharis* rhizosphere	TSA
9	ET52	*Serratia* sp.	*Eleocharis* rhizosphere	TSA
10	ET57	*Pseudomonas monteilii*	*Eleocharis* rhizosphere	TSA
11	ET60	*Azomonas macrocytogenes*	*Eleocharis* rhizosphere	TSA
12	EB3	*Pseudomonas songnenensis*	*Eleocharis* rhizosphere	B-H_amended diesel
13	EB31	*Stenotrophomonas pavanii*	*Eleocharis* rhizosphere	B-H_amended diesel
14	WT4	*Pseudomonas mandelii*	*Salix* rhizosphere	1/10TSA
15	WT8	*Streptomyces atriruber*	*Salix* rhizosphere	1/10TSA
16	WT17	*Pseudomonas kilonensis*	*Salix* rhizosphere	1/10TSA
17	WT22	*Pseudomonas frederiksbergensis*	*Salix* rhizosphere	1/10TSA
18	WT32	*Bacillus megaterium*	*Salix* rhizosphere	1/10TSA
19	WT50	*Pseudomonas frederiksbergensis*	*Salix* rhizosphere	1/10TSA
20	WT56	*Pseudomonas frederiksbergensis*	*Salix* rhizosphere	1/10TSA
21	WB31	*Pseudomonas putida*	*Salix* rhizosphere	B-H_amended diesel
22	EA21	*Pantoea agglomerans*	*Eleocharis* rhizosphere	ACCD

**Table 3:** Chemical and physical characteristics of soils used in this study.

	pH (1:1)	CEC (meq/100 g)	N (g/Kg)	P (Kg/ha)	K (Kg/ha)	Ca (Kg/ha)	Mg (kg/ha)	Mn (PPM)	O.M (%)	Fe (PPM)
Salix rhizosphere	7.4	24.8	0.9	<10	453	7323	2127	34.5	3.5	291.79
*Eleocharis* Rhizosphere	7.3	38.7	2.2	<10	566	13961	1851	61.5	6.2	582.56
Bulk soil	7.4	37.2	2.1	13	565	13121	1952	99.7	7.5	580.77

## Data Availability

Sequencing Data are deposited in GenBank and their accession number are provided within the article.

## Supplementary Materials

The following are available online at https://www.mdpi.com/article/10.3390/plants10101987/s1, Table S1: Taxonomic affiliations of rhizospheric bacteria isolated from *Salix* rhizosphere on different media based on 16S rRNA gene, Table S2: Taxonomic affiliations of rhizospheric bacteria isolated from *Eleocharis* rhizosphere on different media based on 16S rRNA gene, Table S3: Taxonomic affiliations of bacteria isolated from bulk soil on different media based on 16S rRNA gene, Figure S1: Bars indicate the relative abundance of phyla among isolates that possesses different PGP-associated traits in vitro, Figure S2: Qualitative representation of genera among isolates presenting different PGP-associated traits in vitro, Figure S3: Venn diagram of comparison between isolates reported with all five PHC degradation and all five PGP traits, Figure S4: Example of production of siderophore by *Pseudomonas putida* strain ET27 on CAS agar plate, Figure S5: Example of phosphate solubilization by bacterial isolates *Pseudomonas monteilii* strain SB53, *Acinetobacter calcoaceticus* strain SB54 and *Bacillus indicus* strain SB55 as indicated by clear zone on the PDYA-CaP medium.
