# Supplementary material for: Salix purpurea and Eleocharis obtusa Rhizospheres Harbor a Diverse Rhizospheric Bacterial Community Characterized by Hydrocarbons Degradation Potentials and Plant Growth-Promoting Properties
Source: Plants (Basel). 2021 Sep 23;10(10):1987. doi: 10.3390/plants10101987 (PMC8538330; doi:10.3390/plants10101987)
Supplement: Supplementary file 1 [file plants-10-01987-s001.zip › plants-1373585-supplementary.pdf]

Table S1. Taxonomic affiliations of rhizospheric bacteria isolated from *Salix* rhizosphere on different media based on 16S rRNA gene.

Isolate code	Phyla	Family	Closest NCBI relative	SIM (%)	Isolate code	Phyla	Family	Closest NCBI relative	SIM (%)
WB1	Betaproteobacteria	Comamonadaceae	*Variovorax paradoxus*	98	WB41	Actinobacteria	Actinomycetaceae	*Streptomyces phaeochromogenes*	99
WB2	Actinobacteria	Micrococcaceae	*Arthrobacter sulfonivorans*	99	WB42	Firmicutes	Staphylococcaceae	*Staphylococcus warneri*	99
WB3	Actinobacteria	Micrococcaceae	*Arthrobacter nicotinovorans*	99	WB43	Actinobacteria	Micrococcales	*Phycicoccus aerophilus*	98
WB4	Betaproteobacteria	Comamonadaceae	*Variovorax paradoxus*	98	WB44	Actinobacteria	Actinomycetaceae	*Streptomyces umbrinus*	99
WB5	Actinobacteria	Actinomycetaceae	*Streptomyces ederensis*	99	WB45	Actinobacteria	Actinomycetaceae	*Streptomyces umbrinus*	99
WB6	Actinobacteria	Actinomycetaceae	*Streptomyces ederensis*	99	WB46	Actinobacteria	Actinomycetaceae	*Nocardia asteroides*	98
WB7	Actinobacteria	Nocardiaceae	*Nocardia asteroides*	98	WB47	Actinobacteria	Actinomycetaceae	*Streptomyces umbrinus*	98
WB8	Betaproteobacteria	Comamonadaceae	*Variovorax paradoxus*	99	WB48	Actinobacteria	Nocardiaceae	*Nocardia* sp.	98
WB9	Betaproteobacteria	Comamonadaceae	*Variovorax ureilyticus*	99	WB49	Actinobacteria	Nocardioidaceae	*Nocardioides alpinus*	99
WB10	Betaproteobacteria	Comamonadaceae	*Variovorax ureilyticus*	99	WB50	Actinobacteria	Micrococcaceae	*Arthrobacter humicola*	96
WB11	Actinobacteria	Actinomycetaceae	*Streptomyces sp.*	99	WB51	Actinobacteria	Gordoniaceae	*Gordonia* sp.	99
WB12	Betaproteobacteria	Comamonadaceae	*Variovorax paradoxus*	98	WB52	Actinobacteria	Nocardioidaceae	*Nocardioides albus*	98
WB13	Actinobacteria	Micrococcaceae	*Arthrobacter* sp.	97	WB53	Actinobacteria	Actinomycetaceae	*Streptomyces canus*	98
WB14	Actinobacteria	Micrococcaceae	*Arthrobacter* sp.	99	WB54	Actinobacteria	Micrococcales	*Phycicoccus bigeumensis*	99
WB15	Betaproteobacteria	Comamonadaceae	*Variovorax boronicumulans*	98	WB55	Actinobacteria	Actinomycetaceae	*Streptomyces* sp.	98
WB16	Betaproteobacteria	Comamonadaceae	*Variovorax paradoxus*	99	WB56	Actinobacteria	Micrococcaceae	*Pseudarthrobacter oxydans*	98
WB17	Actinobacteria	Micrococcaceae	*Arthrobacter* sp.	97	WB57	Actinobacteria	Mycobacteriaceae	*Mycolicibacterium vanbaalenii*	99
WB18	Betaproteobacteria	Comamonadaceae	*Variovorax paradoxus*	98	WB58	Actinobacteria	Micrococcaceae	*Micromonospora palomenae*	98
WB19	Actinobacteria	Actinomycetaceae	*Streptomyces* sp.	99	WB59	Actinobacteria	Micrococcaceae	*Pseudarthrobacter sulfonivorans*	98
WB20	Actinobacteria	Nocardioidaceae	*Nocardioides albus*	99	WB60	Actinobacteria	Nocardioidaceae	*Nocardioides albus*	99
WB21	Actinobacteria	Pseudonocardiaceae	*Amycolatopsis speibonae*	99	WT1	Firmicutes	Bacillaceae	*Bacillus cereus*	99
WB22	Actinobacteria	Micrococcaceae	*Arthrobacter pascens*	98	WT2	Gammaproteobacteria	Pseudomonadaceae	*Pseudomonas putida*	99
WB23	Betaproteobacteria	Comamonadaceae	*Variovorax paradoxus*	99	WT3	Firmicutes	Paenibacillaceae	*Paenibacillus polysaccharolyticus*	99
WB24	Actinobacteria	Actinomycetaceae	*Streptomyces canus*	99	WT4	Gammaproteobacteria	Pseudomonadaceae	*Pseudomonas mandelii*	98
WB25	Alphaproteobacteria	Sphingomonadaceae	*Sphingomonas sanxanigenens*	97	WT5	Firmicutes	Bacillaceae	*Bacillus cereus*	99
WB26	Actinobacteria	Actinomycetaceae	*Streptomyces umbrinus*	99	WT6	Firmicutes	Bacillaceae	*Bacillus indicus*	99
WB27	Actinobacteria	Actinomycetaceae	*Streptomyces phaeochromogenes*	99	WT7	Actinobacteria	Actinomycetaceae	*Streptomyces griseolus*	96
WB28	Actinobacteria	Nocardioidaceae	*Nocardioides albus*	99	WT8	Actinobacteria	Actinomycetaceae	*Streptomyces atriruber*	97
WB29	Actinobacteria	Actinomycetaceae	*Streptomyces chartreusis*	98	WT9	Actinobacteria	Micrococcaceae	*Streptomyces umbrinus*	99
WB30	Actinobacteria	Nocardioidaceae	*Nocardioides albus*	99	WT10	Firmicutes	Bacillaceae	*Bacillus megaterium*	99
WB31	Gammaproteobacteria	Pseudomonadaceae	*Pseudomonas frederiksbergensis*	98	WT11	Actinobacteria	Actinomycetaceae	*Streptomyces bobili*	98
WB32	Actinobacteria	Nocardioidaceae	*Kribbella aluminosa*	98	WT12	Actinobacteria	Gordoniaceae	*Gordonia amicalis*	98
WB33	Actinobacteria	Micrococcaceae	*Paenarthrobacter nitroguajacolicus*	97	WT13	Actinobacteria	Actinomycetaceae	*Streptomyces pseudovenezuelae*	99
WB34	Actinobacteria	Microbacteriaceae	*Microbacterium oxydans*	99	WT14	Actinobacteria	Actinomycetaceae	*Streptomyces bobili*	98
WB35	Actinobacteria	Nocardioidaceae	*Kribbella sindirgiensis*	98	WT15	Firmicutes	Bacillaceae	*Bacillus aryabhattai*	99
WB36	Actinobacteria	Micrococcaceae	*Pseudarthrobacter oxydans*	98	WT16	Actinobacteria	Micrococcaceae	*Micromonospora halotolerans*	98
WB37	Actinobacteria	Nocardioidaceae	*Kribbella koreensis*	99	WT17	Gammaproteobacteria	Pseudomonadaceae	*Pseudomonas kilonensis*	99
WB38	Actinobacteria	Nocardioidaceae	*Nocardioides* sp.	98	WT18	Actinobacteria	Nocardioidaceae	*Nocardioides albus*	96
WB39	Actinobacteria	Actinomycetaceae	*Streptomyces umbrinus*	99	WT19	Actinobacteria	Micrococcaceae	*Pseudarthrobacter siccitolerans*	96
WB40	Actinobacteria	Micrococcaceae	*Pseudarthrobacter siccitolerans*	98	WT20	Firmicutes	Bacillaceae	*Bacillus indicus*	99

Table S1 Continued.

Isolate code	Phyla	Family	Closest NCBI relative	SIM (%)	Isolate code	Phyla	Family	Closest NCBI relative	SIM (%)
WT21	Alphaproteobacteria	Phyllobacteriaceae	*Mesorhizobium norvegicum*	98	WT54	Actinobacteria	Actinomycetaceae	*Streptomyces bobili*	98
WT22	Gammaproteobacteria	Pseudomonadaceae	*Pseudomonas frederiksbergensis*	97	WT55	Actinobacteria	Actinomycetaceae	*Streptomyces bobili*	99
WT23	Actinobacteria	Micrococcaceae	*Pseudarthrobacter siccitolerans*	95	WT56	Gammaproteobacteria	Pseudomonadaceae	*Pseudomonas frederiksbergensis*	97
WT24	Actinobacteria	Micrococcaceae	*Pseudarthrobacter defluvii*	97	WT57	Gammaproteobacteria	Pseudomonadaceae	*Pseudomonas donghuensis*	99
WT25	Firmicutes	Bacillaceae	*Bacillus simplex*	99	WT58	Actinobacteria	Nocardiaceae	*Rhodococcus degradans*	99
WT26	Actinobacteria	Actinomycetaceae	*Streptomyces griseolus*	99	WT59	Actinobacteria	Actinomycetaceae	*Streptomyces griseolus*	99
WT27	Actinobacteria	Actinomycetaceae	*Streptomyces umbrinus*	99	WT60	Firmicutes	Bacillaceae	*Bacillus thuringiensis*	99
WT28	Firmicutes	Bacillaceae	*Bacillus aryabhattai*	98	WA1	Gammaproteobacteria	Enterobacteriaceae	*Raoultella terrigena*	97
WT29	Firmicutes	Bacillaceae	*Bacillus cereus*	98	WA2	Gammaproteobacteria	Enterobacteriaceae	*Raoultella terrigena*	97
WT30	Firmicutes	Bacillaceae	*Bacillus indicus*	98	WA3	Gammaproteobacteria	Enterobacteriaceae	*Raoultella terrigena*	97
WT31	Firmicutes	Bacillaceae	*Lysinibacillus xylanilyticus*	98	WA4	Gammaproteobacteria	Enterobacteriaceae	*Klebsiella grimontii*	97
WT32	Firmicutes	Bacillaceae	*Bacillus megaterium*	99	WA5	Gammaproteobacteria	Enterobacteriaceae	*Enterobacter cancerogenus*	98
WT33	Actinobacteria	Micrococcaceae	*Mycolicibacterium vanbaalenii*	99	WA6	Gammaproteobacteria	Enterobacteriaceae	*Klebsiella grimontii*	98
WT34	Actinobacteria	Micrococcaceae	*Pseudarthrobacter oxydans*	96	WA7	Gammaproteobacteria	Enterobacteriaceae	*Raoultella terrigena*	98
WT35	Firmicutes	Bacillaceae	*Bacillus thuringiensis*	98	WA8	Gammaproteobacteria	Erwiniaceae	*Pantoea* sp.	98
WT36	Firmicutes	Bacillaceae	*Bacillus indicus*	98	WA9	Gammaproteobacteria	Enterobacteriaceae	*Enterobacter* sp.	99
WT37	Actinobacteria	Micrococcaceae	*Arthrobacter* sp.	98	WA10	Gammaproteobacteria	Enterobacteriaceae	*Klebsiella* sp.	99
WT38	Actinobacteria	Micrococcaceae	*Arthrobacter* sp.	97	WA11	Gammaproteobacteria	Enterobacteriaceae	*Raoultella terrigena*	97
WT39	Actinobacteria	Actinomycetaceae	*Streptomyces atratus*	98	WA12	Gammaproteobacteria	Enterobacteriaceae	*Klebsiella oxytoca*	99
WT40	Actinobacteria	Micrococcaceae	*Paenarthrobacter nitroguajacolicus*	96	WA13	Gammaproteobacteria	Pseudomonadaceae	*Pseudomonas donghuensis*	99
WT41	Betaproteobacteria	Oxalobacteraceae	*Massilia suwonensis*	97	WA14	Gammaproteobacteria	Pseudomonadaceae	*Pseudomonas* donghuensis	98
WT42	Actinobacteria	Actinomycetaceae	*Streptomyces pseudovenezuelae*	98	WA15	Gammaproteobacteria	Xanthomonadaceae	*Stenotrophomonas* sp.	96
WT43	Actinobacteria	Actinomycetaceae	*Streptomyces pseudovenezuelae*	98	WA16	Gammaproteobacteria	Pseudomonadaceae	*Pseudomonas mosselii*	94
WT44	Firmicutes	Bacillaceae	*Bacillus simplex*	99	WA17	Gammaproteobacteria	Pseudomonadaceae	*Pseudomonas plecoglossicida*	98
WT45	Alphaproteobacteria	Caulobacteraceae	*Caulobacter rhizosphaerae*	96	WA18	Gammaproteobacteria	Enterobacteriaceae	*Raoultella terrigena*	98
WT46	Actinobacteria	Nocardiaceae	*Rhodococcus degradans*	95	WA19	Gammaproteobacteria	Enterobacteriaceae	*Citrobacter freundii*	98
WT47	Firmicutes	Bacillaceae	*Bacillus indicus*	99	WA20	Gammaproteobacteria	Enterobacteriaceae	*Enterobacter cancerogenus*	98
WT48	Actinobacteria	Micrococcaceae	*Arthrobacter* sp.	98	WA21	Gammaproteobacteria	Pseudomonadaceae	*Pseudomonas fluorescens*	99
WT49	Actinobacteria	Gordoniaceae	*Gordonia amicalis*	99	WA22	Betaproteobacteria	Comamonadaceae	*Variovorax boronicumulans*	98
WT50	Gammaproteobacteria	Pseudomonadaceae	*Pseudomonas frederiksbergensis*	98	WA23	Actinobacteria	Pseudonocardiaceae	*Amycolatopsis azurea*	98
WT51	Firmicutes	Bacillaceae	*Bacillus indicus*	99	WA24	Gammaproteobacteria	Pseudomonadaceae	*Pseudomonas kilonensis*	99
WT52	Actinobacteria	Actinomycetaceae	*Streptomyces bobili*	98	WA25	Gammaproteobacteria	Pseudomonadaceae	*Pseudomonas brassicacearum*	99
WT53	Actinobacteria	Actinomycetaceae	*Streptomyces bobili*	99	WA28	Betaproteobacteria	Comamonadaceae	*Variovorax paradoxus*	99

[1] Isolate code refers to the environmental niche and isolation medium from which the isolate came. The first letter (W) indicates that the isolate was from willow rhizosphere. The second letter indicates the isolation media used to cultivates bacterial isolates (B= Bushnell-Haas medium amended with 1 % diesel, as the sole carbon and energy source, T= One-tenth-strength Trypticase Soy Agar (TSA) medium and A= DF-ACC agar. The Isolate numbers was randomly assigned.

Table S2. Taxonomic affiliations of rhizospheric bacteria isolated from *Eleocharis* rhizosphere on different media based on 16S rRNA gene.

Isolate code	Phyla	Family	Closest NCBI relative	SIM (%)	Isolate code	Phyla	Family	Closest NCBI relative	SIM (%)
EB1	Gammaproteobacteria	Pseudomonadaceae	*Pseudomonas helmanticensis*	99	EB41	Unidentified	Unidentified	*Unidentified bacterium*	98
EB2	Firmicutes	Bacillaceae	*Bacillus indicus*	99	EB42	Alphaproteobacterial	Sphingomonadaceae	*Sphingopyxis soli*	98
EB3	Gammaproteobacteria	Pseudomonadaceae	*Pseudomonas songnenensis*	98	EB43	Gammaproteobacteria	Pseudomonadaceae	*Pseudomonas entomophila*	99
EB4	Gammaproteobacteria	Pseudomonadaceae	*Pseudomonas geniculate*	98	EB44	Gammaproteobacteria	Pseudomonadaceae	*Pseudomonas mosselii*	98
EB5	Betaproteobacteria	Comamonadaceae	*Variovorax boronicumulans*	98	EB45	Alphaproteobacteria	Xanthobacteraceae	*Azorhizobium* sp.	97
EB6	Betaproteobacteria	Burkholderiaceae	*Chitinimonas taiwanensis*	98	EB46	Alphaproteobacteria	Rhizobiaceae	*Rhizobium petrolearium*	99
EB7	Gammaproteobacteria	Pseudomonadaceae	*Pseudomonas alcaligenes*	98	EB47	Alphaproteobacteria	Bradyrhizobiaceae	*Bosea thiooxidans*	97
EB8	Actinobacteria	Microbacteriaceae	*Microbacterium pumilum*	99	EB48	Betaproteobacteria	Alcaligenaceae	*Achromobacter spanius*	98
EB9	Gammaproteobacteria	Pseudomonadaceae	*Pseudomonas mosselii*	97	EB49	Actinobacteria	Nocardiaceae	*Rhodococcus ruber*	98
EB10	Actinobacteria	Actinomycetaceae	*Streptomyces stelliscabiei*	99	EB50	Unidentified	Unidentified	*Unidentified bacterium*	98
EB11	Actinobacteria	Microbacteriaceae	*Microbacterium lacus*	98	EB51	Alphaproteobacteria	Xanthobacteraceae	*Azorhizobium doebereinerae*	99
EB12	Gammaproteobacteria	Pseudomonadaceae	*Pseudomonas mosselii*	97	EB52	Gammaproteobacteria	Pseudomonadaceae	*Pseudomonas stutzeri*	98
EB13	Gammaproteobacteria	Moraxellaceae	*Acinetobacter junii*	99	EB53	Actinobacteria	Microbacteriaceae	*Microbacterium oxydans*	99
EB14	Gammaproteobacteria	Pseudomonadaceae	*Pseudomonas mosselii*	96	EB54	Gammaproteobacteria	Pseudomonadaceae	*Pseudomonas stutzeri*	98
EB15	Gammaproteobacteria	Pseudomonadaceae	*Pseudomonas plecoglossicida*	99	EB55	Gammaproteobacteria	Pseudomonadaceae	*Pseudomonas putida*	98
EB16	Gammaproteobacteria	Pseudomonadaceae	*Pseudomonas plecoglossicida*	99	EB56	Betaproteobacteria	Comamonadaceae	*Delftia lacustris*	99
EB17	Actinobacteria	Microbacteriaceae	*Microbacterium oxydans*	98	EB57	Actinobacteria	Microbacteriaceae	*Agromyces indicus*	98
EB18	Betaproteobacteria	Comamonadaceae	*Acidovorax facilis*	98	EB58	Gammaproteobacteria	Xanthomonadaceae	*Pseudoxanthomonas japonensis*	99
EB19	Gammaproteobacteria	Xanthomonadaceae	*Dyella ginsengisoli*	98	EB59	Gammaproteobacteria	Pseudomonadaceae	*Pseudomonas mosselii*	98
EB20	Gammaproteobacteria	Xanthomonadaceae	*Stenotrophomonas nitritireducens*	96	EB60	Gammaproteobacteria	Pseudomonadaceae	*Pseudomonas silesiensis*	99
EB21	Gammaproteobacteria	Aeromonadaceae	*Aeromonas salmonicida*	99	ET1	Betaproteobacteria	Burkholderiaceae	*Chitinimonas taiwanensis*	98
EB22	Gammaproteobacteria	Pseudomonadaceae	*Pseudomonas mosselii*	97	ET2	Gammaproteobacteria	Xanthomonadaceae	*Pseudoxanthomonas mexicana*	99
EB23	Gammaproteobacteria	Pseudomonadaceae	*Pseudomonas putida*	98	ET3	Unidentified	Unidentified	*Unidentified bacterium*	98
EB24	Actinobacteria	Microbacteriaceae	*Microbacterium lacus*	97	ET4	Gammaproteobacteria	Xanthomonadaceae	*Pseudoxanthomonas spadix*	99
EB25	Gammaproteobacteria	Aeromonadaceae	*Aeromonas salmonicida*	99	ET5	Actinobacteria	Microbacteriaceae	*Microbacterium testaceum*	98
EB26	Gammaproteobacteria	Aeromonadaceae	*Aeromonas hydrophila*	98	ET6	Actinobacteria	Micrococcales	*Lysinimonas* sp.	99
EB27	Actinobacteria	Microbacteriaceae	*Microbacterium kitamiense*	99	ET7	Bacteroidetes	Flavobacteriaceae	*Chryseobacterium candidae*	96
EB28	Gammaproteobacteria	Pseudomonadaceae	*Pseudomonas plecoglossicida*	99	ET8	Alphaproteobacteria	Rhizobiaceae	*Rhizobium selenitireducens*	96
EB29	Gammaproteobacteria	Aeromonadaceae	*Aeromonas sobria*	99	ET9	Alphaproteobacteria	Rhizobiaceae	*Rhizobium rosettiformans*	98
EB30	Gammaproteobacteria	Pseudomonadaceae	*Pseudomonas fluorescens*	99	ET10	Alphaproteobacteria	Rhizobiaceae	*Rhizobium selenitireducens*	99
EB31	Gammaproteobacteria	Xanthomonadaceae	*Stenotrophomonas pavanii*	98	ET11	Firmicutes	Bacillaceae	*Bacillus indicus*	99
EB32	Gammaproteobacteria	Pseudomonadaceae	*Pseudomonas putida*	99	ET12	Firmicutes	Bacillaceae	*Bacillus indicus*	96
EB33	Gammaproteobacteria	Pseudomonadaceae	*Pseudomonas plecoglossicida*	99	ET13	Firmicutes	Bacillaceae	*Bacillus indicus*	99
EB34	Gammaproteobacteria	Pseudomonadaceae	*Pseudomonas fildesensis*	98	ET14	Gammaproteobacteria	Xanthomonadaceae	*Pseudoxanthomonas spadix*	99
EB35	Betaproteobacteria	Comamonadaceae	*Comamonas odontotermitis*	99	ET15	Alphaproteobacteria	Sphingomonadaceae	*Sphingopyxis soli*	98
EB36	Gammaproteobacteria	Pseudomonadaceae	*Pseudomonas plecoglossicida*	98	ET16	Firmicutes	Bacillaceae	*Bacillus aquimaris*	98
EB37	Actinobacteria	Micrococcales	*Lysinimonas* sp.	99	ET17	Bacteroidetes	Flavobacteriaceae	*Flavihumibacter cheonanensis*	99
EB38	Betaproteobacteria	Comamonadaceae	*Delftia lacustris*	99	ET18	Bacteroidetes	Flavobacteriaceae	*Chryseobacterium elymi*	97
EB39	Actinobacteria	Microbacteriaceae	*Microbacterium proteolyticum*	97	ET19	Actinobacteria	Microbacteriaceae	*Microbacterium saccharophilum*	95
EB40	Actinobacteria	Microbacteriaceae	*Microbacterium saccharophilum*	98	ET20	Unidentified	Unidentified	*Unidentified bacterium*	97

Table S2 Continued.

Isolate code	Phyla	Family	Closest NCBI relative	SIM (%)	Isolate code	Phyla	Family	Closest NCBI relative	SIM (%)
ET21	Alphaproteobacteria	Sphingomonadaceae	*Sphingomonas dokdonensis*	96	ET54	Gammaproteobacteria	Pseudomonadaceae	*Pseudomonas mosselii*	99
ET22	Gammaproteobacteria	Xanthomonadaceae	*Pseudoxanthomonas spadix*	98	ET55	Alphaproteobacteria	Bradyrhizobiaceae	*Bosea thiooxidans*	98
ET23	Unidentified	Unidentified	*Unidentified bacterium*	98	ET56	Alphaproteobacteria	Xanthobacteraceae	*Brevundimonas denitrificans*	98
ET24	Unidentified	Unidentified	*Unidentified bacterium*	98	ET57	Gammaproteobacteria	Pseudomonadaceae	*Pseudomonas monteilii*	99
ET25	Firmicutes	Bacillaceae	*Bacillus marisflavi*	99	ET58	Firmicutes	Bacillaceae	*Exiguobacterium acetylicum*	99
ET26	Unidentified	Unidentified	*Unidentified bacterium*	98	ET59	Firmicutes	Bacillaceae	*Exiguobacterium undae*	98
ET27	Gammaproteobacteria	Pseudomonadaceae	*Pseudomonas plecoglossicida*	99	ET60	Gammaproteobacteria	Pseudomonadaceae	*Azomonas macrocytogenes*	98
ET28	Actinobacteria	Microbacteriaceae	*Microbacterium testaceum*	97	EA1	Gammaproteobacteria	Enterobacteriaceae	*Klebsiella oxytoca*	98
ET29	Gammaproteobacteria	Xanthomonadaceae	*Pseudoxanthomonas spadix*	98	EA2	Gammaproteobacteria	Enterobacteriaceae	*Klebsiella oxytoca*	98
ET30	Gammaproteobacteria	Pseudomonadaceae	*Pseudomonas fildesensis*	99	EA3	Gammaproteobacteria	Enterobacteriaceae	*Klebsiella oxytoca*	99
ET31	Alphaproteobacteria	Rhodospirillaceae	*Rhodospirillum* sp.	97	EA4	Gammaproteobacteria	Enterobacteriaceae	*Klebsiella oxytoca*	99
ET32	Betaproteobacteria	Comamonadaceae	*Variovorax paradoxus*	99	EA5	Gammaproteobacteria	Enterobacteriaceae	*Klebsiella oxytoca*	98
ET33	Betaproteobacteria	Comamonadaceae	*Delftia lacustris*	99	EA6	Gammaproteobacteria	Enterobacteriaceae	*Klebsiella oxytoca*	98
ET34	Actinobacteria	Microbacteriaceae	*Agromyces tropicus*	97	EA7	Gammaproteobacteria	Enterobacteriaceae	*Klebsiella variicola*	99
ET35	Actinobacteria	Microbacteriaceae	*Microbacterium oxydans*	99	EA8	Gammaproteobacteria	Enterobacteriaceae	*Klebsiella michiganensis*	97
ET36	Firmicutes	Bacillaceae	*Bacillus indicus*	99	EA9	Gammaproteobacteria	Enterobacteriaceae	*Enterobacter cancerogenus*	97
ET37	Gammaproteobacteria	Chromatiaceae	*Pararheinheimera arenilitoris*	97	EA10	Gammaproteobacteria	Enterobacteriaceae	*Klebsiella oxytoca*	98
ET38	Actinobacteria	Nocardiaceae	*Rhodococcus ruber*	98	EA11	Gammaproteobacteria	Enterobacteriaceae	*Klebsiella oxytoca*	98
ET39	Alphaproteobacteria	Sphingomonadaceae	*Sphingopyxis soli*	96	EA12	Gammaproteobacteria	Enterobacteriaceae	*Klebsiella oxytoca*	97
ET40	Gammaproteobacteria	Pseudomonadaceae	*Pseudomonas chengduensis*	97	EA13	Bacteroidetes	Flavobacteriaceae	*Myroides odoratimimus*	97
ET41	Gammaproteobacteria	Pseudomonadaceae	*Pseudomonas mosselii*	99	EA14	Gammaproteobacteria	Moraxellaceae	*Acinetobacter pittii*	99
ET42	Gammaproteobacteria	Pseudomonadaceae	*Pseudomonas mendocina*	99	EA15	Gammaproteobacteria	Moraxellaceae	*Acinetobacter johnsonii*	99
ET43	Gammaproteobacteria	Yersiniaceae	*Serratia* sp.	97	EA16	Bacteroidetes	Flavobacteriaceae	*Empedobacter tilapiae*	99
ET44	Gammaproteobacteria	Yersiniaceae	*Serratia* sp.	98	EA17	Gammaproteobacteria	Moraxellaceae	*Acinetobacter calcoaceticus*	99
ET45	Gammaproteobacteria	Pseudomonadaceae	*Pseudomonas fluorescens*	99	EA18	Bacteroidetes	Flavobacteriaceae	*Myroides odoratus*	98
ET46	Gammaproteobacteria	Yersiniaceae	*Serratia* sp.	97	EA19	Gammaproteobacteria	Enterobacteriaceae	*Enterobacter* sp.	97
ET47	Firmicutes	Bacillaceae	*Bacillus indicus*	99	EA20	Bacteroidetes	Flavobacteriaceae	*Myroides odoratimimus*	97
ET48	Gammaproteobacteria	Pseudomonadaceae	*Pseudomonas mosselii*	98	EA21	Gammaproteobacteria	Enterobacteriaceae	*Pantoea agglomerans*	98
ET49	Gammaproteobacteria	Enterobacteriaceae	*Enterobacter cancerogenus*	98	EA22	Gammaproteobacteria	Pseudomonadaceae	*Pseudomonas koreensis*	98
ET50	Gammaproteobacteria	Pseudomonadaceae	*Pseudomonas putida*	99	EA23	Actinobacteria	Mycobacteriaceae	*Mycobacterium aquiterrae*	99
ET51	Gammaproteobacteria	Yersiniaceae	*Serratia* sp.	97	EA24	Actinobacteria	Microbacteriaceae	*Microbacterium oxydans*	99
ET52	Gammaproteobacteria	Yersiniaceae	*Serratia* sp.	98	EA25	Actinobacteria	Mycobacteriaceae	*Mycolicibacterium vanbaalenii*	99
ET53	Gammaproteobacteria	Pseudomonadaceae	*Pseudomonas mosselii*	98	EA27	Actinobacteria	Mycobacteriaceae	*Mycolicibacterium vanbaalenii*	98

[1] Isolate code refers to the environmental niche and isolation medium from which the isolate came. The first letter (E) indicates that the isolate where from *Eleocharis* rhizosphere. The second letter indicates the isolation media used to cultivates bacterial isolates (B= Bushnell-Haas medium amended with 1 % diesel, as the sole carbon and energy source, T= One-tenth-strength Trypticase Soy Agar (TSA) medium and A= DF-ACC agar. The Isolate numbers was randomly assigned.

Table S3. Taxonomic affiliations of bacteria isolated from bulk soil on different media based on 16S rRNA gene.

Isolate code	Phyla	Family	Closest NCBI relative	SIM (%)	Isolate code	Phyla	Family	Closest NCBI relative	SIM (%)
SB1	Gammaproteobacteria	Moraxellaceae	*Acinetobacter calcoaceticus*	99	SB41	Gammaproteobacteria	Moraxellaceae	*Acinetobacter calcoaceticus*	99
SB2	Gammaproteobacteria	Moraxellaceae	*Acinetobacter johnsonii*	99	SB42	Gammaproteobacteria	Pseudomonadaceae	*Pseudomonas stutzeri*	96
SB3	Firmicutes	Bacillaceae	*Bacillus toyonensis*	99	SB43	Alphaproteobacteria	Sphingomonadaceae	*Sphingomonas taxi*	99
SB4	Actinobacteria	Microbacteriaceae	*Microbacterium oxydans*	97	SB44	Gammaproteobacteria	Pseudomonadaceae	*Pseudomonas hunanensis*	98
SB5	Gammaproteobacteria	Moraxellaceae	*Acinetobacter johnsonii*	98	SB45	Gammaproteobacteria	Pseudomonadaceae	*Pseudomonas mosselii*	99
SB6	Actinobacteria	Bogoriellaceae	*Georgenia muralis*	95	SB46	Actinobacteria	Microbacteriaceae	*Microbacterium lacus*	98
SB7	Gammaproteobacteria	Moraxellaceae	*Acinetobacter johnsonii*	99	SB47	Gammaproteobacteria	Xanthomonadaceae	*Pseudoxanthomonas spadix*	98
SB8	Actinobacteria	Microbacteriaceae	*Microbacterium lacus*	95	SB48	Gammaproteobacteria	Pseudomonadaceae	*Pseudomonas* sp.	99
SB9	Gammaproteobacteria	Moraxellaceae	*Acinetobacter johnsonii*	99	SB49	Betaproteobacteria	Oxalobacteraceae	*Massilia oculi*	98
SB10	Actinobacteria	Nocardiaceae	*Rhodococcus erythropolis*	98	SB50	Alphaproteobacteria	Sphingomonadaceae	*Sphingobium yanoikuyae*	98
SB11	Betaproteobacteria	Oxalobacteraceae	*Massilia oculi*	99	SB51	Unidentified	Unidentified	Unidentified bacterium	98
SB12	Gammaproteobacteria	Pseudomonadaceae	*Pseudomonas* sp.	98	SB52	Unidentified	Unidentified	Unidentified bacterium	99
SB13	Unidentified	Unidentified	Unidentified bacterium	99	SB53	Gammaproteobacteria	Pseudomonadaceae	*Pseudomonas monteilii*	98
SB14	Actinobacteria	Microbacteriaceae	*Microbacterium oxydans*	98	SB54	Gammaproteobacteria	Moraxellaceae	*Acinetobacter calcoaceticus*	99
SB15	Gammaproteobacteria	Xanthomonadaceae	*Pseudoxanthomonas spadix*	98	SB55	Gammaproteobacteria	Moraxellaceae	*Acinetobacter calcoaceticus*	99
SB16	Gammaproteobacteria	Pseudomonadaceae	*Pseudomonas putida*	98	SB56	Firmicutes	Bacillaceae	*Bacillus indicus*	99
SB17	Gammaproteobacteria	Moraxellaceae	*Acinetobacter calcoaceticus*	98	SB57	Betaproteobacteria	Oxalobacteraceae	*Massilia* sp.	99
SB18	Actinobacteria	Microbacteriaceae	*Agromyces indicus*	98	SB58	Gammaproteobacteria	Pseudomonadaceae	*Pseudomonas stutzeri*	99
SB19	Gammaproteobacteria	Pseudomonadaceae	*Pseudomonas putida*	98	SB59	Gammaproteobacteria	Pseudomonadaceae	*Pseudomonas mosselii*	99
SB20	Gammaproteobacteria	Moraxellaceae	*Acinetobacter johnsonii*	98	SB60	Gammaproteobacteria	Moraxellaceae	*Acinetobacter* sp.	98
SB21	Unidentified	Unidentified	Unidentified bacterium	95	ST1	Gammaproteobacteria	Xanthomonadaceae	*Stenotrophomonas nitritireducens*	99
SB22	Alphaproteobacteria	Rhizobiaceae	*Rhizobium* sp.	99	ST2	Firmicutes	Bacillaceae	*Bacillus idriensis*	99
SB23	Unidentified	Unidentified	Unidentified bacterium	97	ST3	Actinobacteria	Microbacteriaceae	*Agromyces indicus*	95
SB24	Gammaproteobacteria	Xanthomonadaceae	*Stenotrophomonas chelatiphaga*	98	ST4	Actinobacteria	Nocardiaceae	*Rhodococcus ruber*	97
SB25	Firmicutes	Bacillaceae	*Bacillus siamensis*	99	ST5	Betaproteobacteria	Oxalobacteraceae	*Massilia* sp.	98
SB26	Alphaproteobacteria	Paracoccus	*Paracoccus* sp.	99	ST6	Gammaproteobacteria	Chromatiaceae	*Rheinheimera arenilitoris*	98
SB27	Actinobacteria	Nocardiaceae	*Rhodococcus ruber*	98	ST7	Actinobacteria	Microbacteriaceae	*Agromyces indicus*	96
SB28	Unidentified	Unidentified	Unidentified bacterium	99	ST8	Gammaproteobacteria	Xanthomonadaceae	*Pseudoxanthomonas* sp.	97
SB29	Gammaproteobacteria	Pseudomonadaceae	Pseudomonas kunmingensis	98	ST9	Alphaproteobacteria	Caulobacteraceae	*Brevundimonas nasdae*	97
SB30	Firmicutes	Paenibacillaceae	*Brevibacillus nitrificans*	97	ST10	Unidentified	Unidentified	Unidentified bacterium	98
SB31	Gammaproteobacteria	Xanthomonadaceae	*Stenotrophomonas tumulicola*	99	ST11	Unidentified	Unidentified	Unidentified bacterium	97
SB32	Actinobacteria	Microbacteriaceae	*Microbacterium hatanonis*	98	ST12	Gammaproteobacteria	Xanthomonadaceae	*Pseudoxanthomonas spadix*	97
SB33	Gammaproteobacteria	Moraxellaceae	*Acinetobacter* sp.	98	ST13	Gammaproteobacteria	Xanthomonadaceae	*Pseudoxanthomonas spadix*	98
SB34	Gammaproteobacteria	Moraxellaceae	*Acinetobacter calcoaceticus*	99	ST14	Betaproteobacteria	Comamonadaceae	*Hydrogenophaga* sp.	98
SB35	Gammaproteobacteria	Moraxellaceae	*Acinetobacter calcoaceticus*	99	ST15	Gammaproteobacteria	Pseudomonadaceae	*Pseudomonas* sp.	97
SB36	Gammaproteobacteria	Moraxellaceae	*Acinetobacter pittii*	98	ST16	Firmicutes	Bacillaceae	*Bacillus cibi*	99
SB37	Gammaproteobacteria	Pseudomonadaceae	*Pseudomonas fulva*	99	ST17	Gammaproteobacteria	Rhodanobacteraceae	*Luteibacter jiangsuensis*	97
SB38	Gammaproteobacteria	Pseudomonadaceae	*Pseudomonas stutzeri*	98	ST18	Gammaproteobacteria	Bacillaceae	*Bacillus aquimaris*	96
SB39	Actinobacteria	Microbacteriaceae	*Microbacterium oxydans*	98	ST19	Gammaproteobacteria	Xanthomonadaceae	*Pseudoxanthomonas spadix*	97
SB40	Actinobacteria	Microbacteriaceae	*Microbacterium oxydans*	98	ST20	Bacteroidetes	Flavobacteriaceae	*Chryseobacterium halperniae*	95

Table S3 Continued.

Isolate code	Phyla	Family	Closest NCBI relative	SIM (%)	Isolate code	Phyla	Family	Closest NCBI relative	SIM (%)
ST21	Gammaproteobacteria	Rhodanobacteraceae	*Dyella ginsengisoli*	99	ST54	Firmicutes	Bacillaceae	*Bacillus cereus*	97
ST22	Unidentified	Unidentified	Unidentified	98	ST55	Unidentified	Unidentified	Unidentified bacterium	96
ST23	Actinobacteria	Nocardiaceae	*Rhodococcus ruber*	99	ST56	Gammaproteobacteria	Xanthomonadaceae	*Pseudoxanthomonas spadix*	98
ST24	Gammaproteobacteria	Rhodanobacteraceae	*Dyella ginsengisoli*	98	ST57	Gammaproteobacteria	Xanthomonadaceae	*Pseudoxanthomonas* sp.	95
ST25	Gammaproteobacteria	Xanthomonadaceae	*Stenotrophomonas nitritireducens*	98	ST58	Gammaproteobacteria	Xanthomonadaceae	*Pseudoxanthomonas spadix*	99
ST26	Actinobacteria	Microbacteriaceae	*Agrococcus* sp.	99	ST59	Actinobacteria	Nocardiaceae	*Rhodococcus* sp.	95
ST27	Firmicutes	Bacillaceae	*Bacillus thuringiensis*	98	ST60	Gammaproteobacteria	Xanthomonadaceae	*Pseudoxanthomonas* sp.	98
ST28	Unidentified	Unidentified	Unidentified bacterium	98	SA1	Gammaproteobacteria	Moraxellaceae	*Acinetobacter pittii*	98
ST29	Unidentified	Unidentified	Unidentified bacterium	98	SA2	Gammaproteobacteria	Enterobacteriaceae	*Klebsiella variicola*	97
ST30	Firmicutes	Bacillaceae	*Bacillus idriensis*	99	SA3	Gammaproteobacteria	Enterobacteriaceae	*Klebsiella variicola*	97
ST31	Actinobacteria	Microbacteriaceae	*Microbacterium natoriense*	99	SA4	Gammaproteobacteria	Enterobacteriaceae	*Klebsiella variicola*	98
ST32	Unidentified	Unidentified	*Unidentified bacterium*	98	SA5	Gammaproteobacteria	Enterobacteriaceae	*Klebsiella* variicola	97
ST33	Firmicutes	Bacillaceae	*Bacillus aryabhattai*	99	SA6	Gammaproteobacteria	Enterobacteriaceae	*Citrobacter freundii*	98
ST34	Actinobacteria	Nocardiaceae	*Rhodococcus erythropolis*	99	SA7	Gammaproteobacteria	Enterobacteriaceae	*Pantoea agglomerans*	96
ST35	Alphaproteobacteria	Caulobacteraceae	*Brevundimonas alba*	99	SA8	Gammaproteobacteria	Enterobacteriaceae	*Klebsiella oxytoca*	98
ST36	Unidentified	Unidentified	Unidentified bacterium	96	SA9	Gammaproteobacteria	Pseudomonadaceae	*Pseudomonas plecoglossicida*	98
ST37	Actinobacteria	Microbacteriaceae	*Microbacterium* sp.	99	SA10	Gammaproteobacteria	Enterobacteriaceae	*Enterobacter* sp.	98
ST38	Firmicutes	Bacillaceae	*Bacillus aquimaris*	98	SA11	Gammaproteobacteria	Enterobacteriaceae	*Klebsiella variicola*	99
ST39	Gammaproteobacteria	Xanthomonadaceae	*Stenotrophomonas nitritireducens*	97	SA12	Gammaproteobacteria	Enterobacteriaceae	*Klebsiella variicola*	99
ST40	Firmicutes	Bacillaceae	*Exiguobacterium* sp.	98	SA13	Bacteroidetes	Flavobacteriaceae	*Empedobacter tilapiae*	99
ST41	Bacteroidetes	Flavobacteriaceae	*Chryseobacterium elymi*	96	SA14	Alphaproteobacteria	Sphingomonadaceae	*Sphingobacterium* sp.	98
ST42	Firmicutes	Bacillaceae	*Bacillus megaterium*	99	SA15	Gammaproteobacteria	Xanthomonadaceae	*Stenotrophomonas maltophilia*	97
ST43	Gammaproteobacteria	Chromatiaceae	*Rheinheimera arenilitoris*	96	SA16	Bacteroidetes	Flavobacteriaceae	*Myroides odoratus*	98
ST44	Gammaproteobacteria	Xanthomonadaceae	*Stenotrophomonas* sp.	99	SA17	Bacteroidetes	Flavobacteriaceae	*Myroides odoratus*	98
ST45	Actinobacteria	Gordoniaceae	*Gordonia amicalis*	99	SA18	Bacteroidetes	Flavobacteriaceae	*Empedobacter tilapiae*	99
ST46	Bacteroidetes	Flavobacteriaceae	*Chryseobacterium elymi*	97	SA19	Gammaproteobacteria	Enterobacteriaceae	*Citrobacter freundii*	98
ST47	Gammaproteobacteria	Rhodanobacteraceae	*Dyella ginsengisoli*	99	SA20	Gammaproteobacteria	Xanthomonadaceae	*Stenotrophomonas maltophilia*	99
ST48	Gammaproteobacteria	Xanthomonadaceae	*Pseudoxanthomonas spadix*	99	SA21	Firmicutes	Staphylococcaceae	*Staphylococcus capitis*	99
ST49	Gammaproteobacteria	Pseudomonadaceae	*Pseudomonas alcaligenes*	97	SA22	Actinobacteria	Mycobacteriaceae	*Mycolicibacterium vanbaalenii*	99
ST50	Gammaproteobacteria	Pseudomonadaceae	*Pseudoxanthomonas spadix*	99	SA23	Actinobacteria	Nocardiaceae	*Rhodococcus ruber*	97
ST51	Firmicutes	Bacillaceae	*Bacillus indicus*	99	SA24	Actinobacteria	Mycobacteriaceae	*Mycolicibacterium vanbaalenii*	98
ST52	Alphaproteobacteria	Sphingomonadaceae	*Sphingopyxis soli*	99	SA25	Actinobacteria	Nocardiaceae	*Rhodococcus ruber*	98
ST53	Firmicutes	Bacillaceae	*Bacillus indicus*	99	SA26	Actinobacteria	Mycobacteriaceae	*Mycolicibacterium vanbaalenii*	99

[1] Isolate code refers to the environmental niche and isolation medium from which the isolate came. The first letter (S) indicates that the bacterial isolate where isolated from bulk soil. The second letter indicates the isolation media used to cultivates bacterial isolates (B= Bushnell-Haas medium amended with 1 % diesel, as the sole carbon and energy source, T= One-tenth-strength Trypticase Soy Agar (TSA) medium and A= DF-ACC agar. The Isolate numbers was randomly assigned.

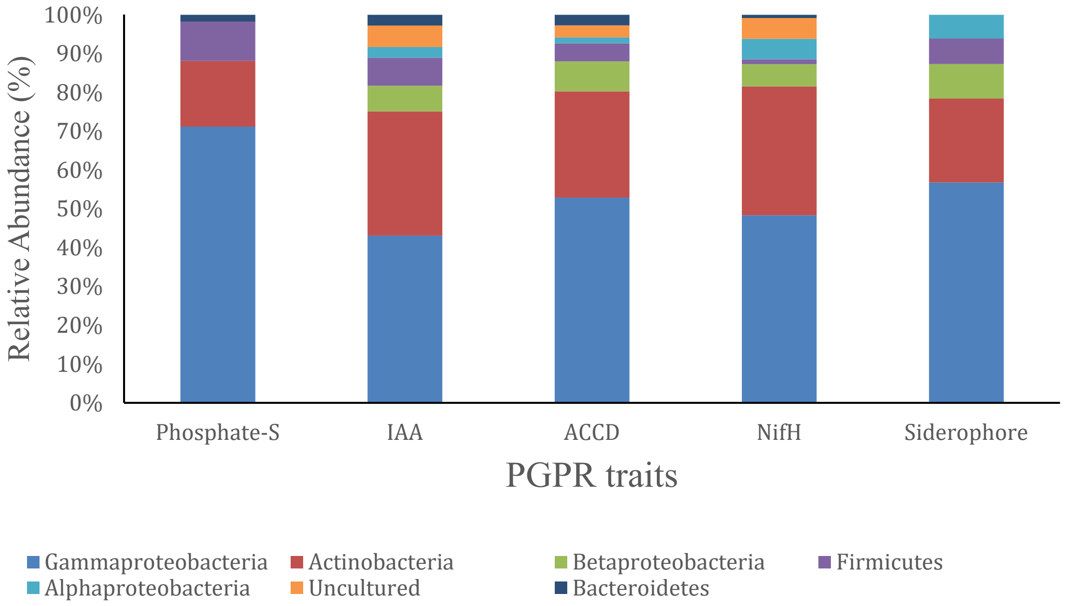

Figure S1. Bars indicate the relative abundance of phyla among isolates that possesses different PGP-associated traits *in vitro.*

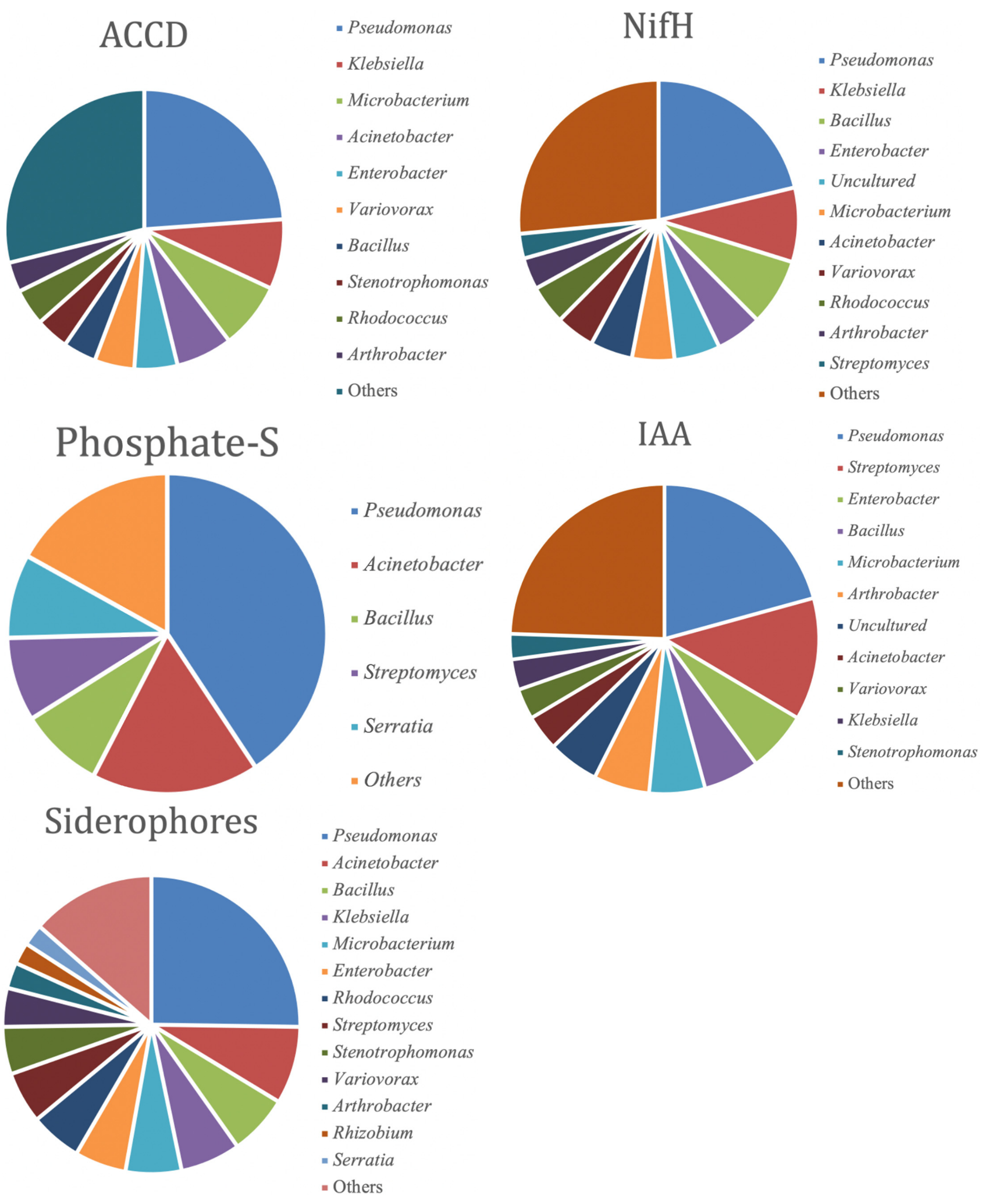

Figure S2 Qualitative representation of genera among isolates presenting different PGP-associated traits *in vitro*.

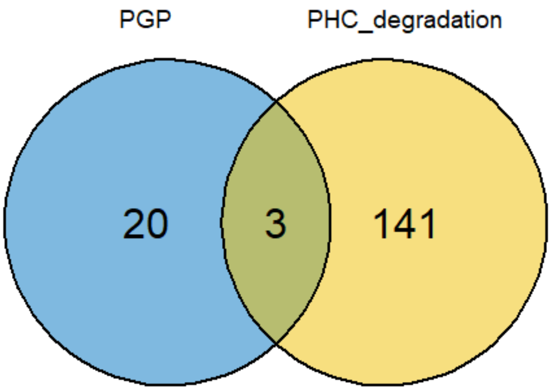

Figure S3. Venn diagram of comparison between isolates reported with all five PHC degradation and all five PGP traits.

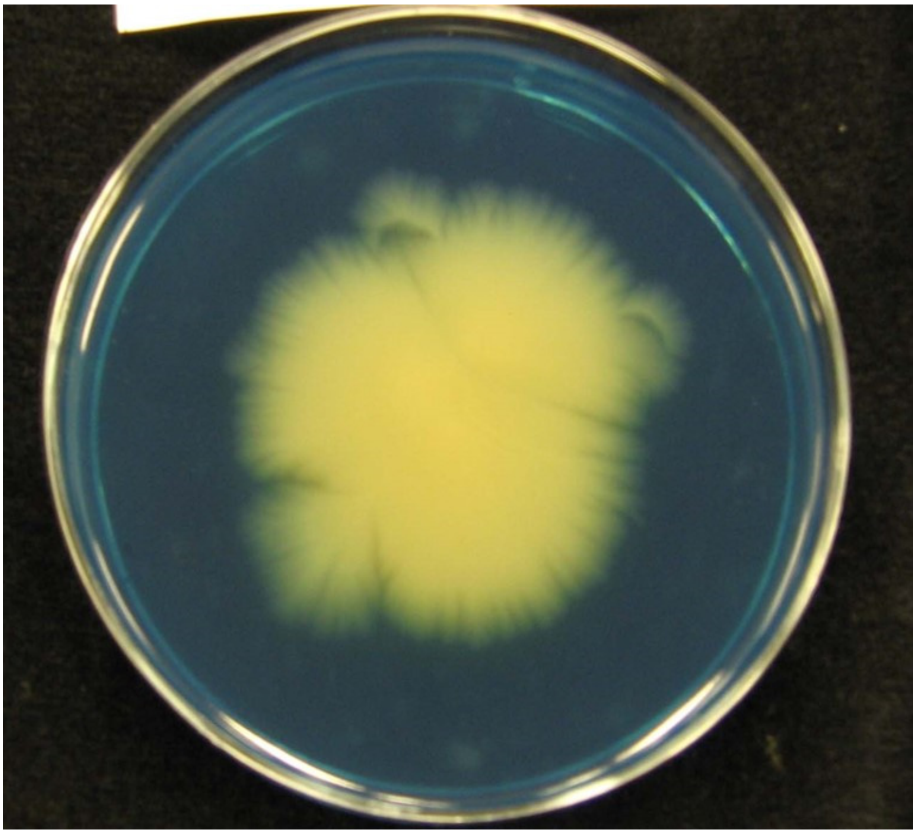

Figure S4. Example of production of siderophore by *Pseudomonas putida* strain ET27 on CAS agar plate.

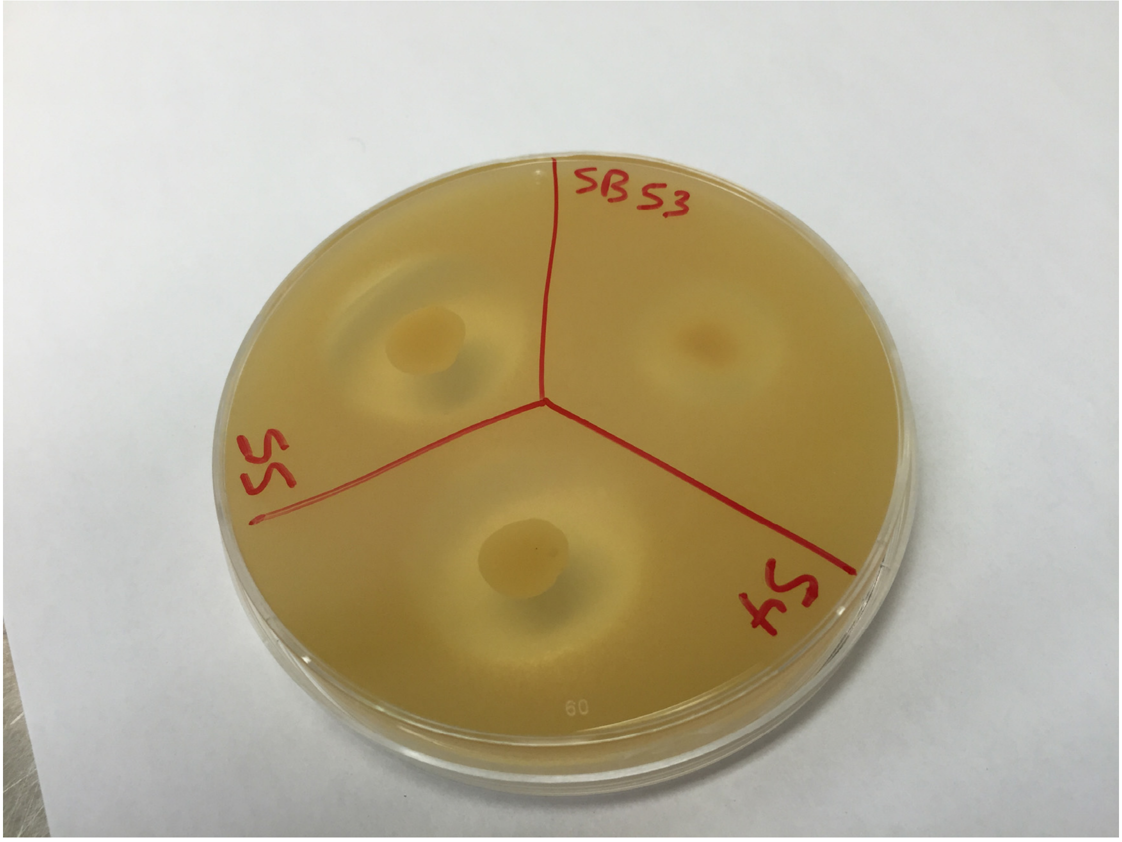

Figure S5. Example of phosphate solubilization by bacterial isolates *Pseudomonas monteilii* strain SB53, *Acinetobacter calcoaceticus* strain SB54 and *Bacillus indicus* strain SB55 as indicated by clear zone on the PDYA-CaP medium.
